# On the potential origin of the zygote-like cancer stem cell with a focus on fusion for cell rescue

**DOI:** 10.3389/fcell.2026.1797239

**Published:** 2026-04-17

**Authors:** Cosmin Andrei Cismaru, Sergiu Chira, George Adrian Calin, Romana Netea-Maier, Ioana Berindan-Neagoe

**Affiliations:** 1 Department of Genomics, Institute of Biomedical Research – MEDFUTURE, “Iuliu Hațieganu” University of Medicine and Pharmacy, Cluj-Napoca, Romania; 2 Department of Translational Molecular Pathology, The University of Texas MD Anderson Cancer Center, Houston, TX, United States; 3 Center for RNA Interference and Non-Coding RNAs, The University of Texas MD Anderson Cancer Center, Houston, TX, United States; 4 Department of Internal Medicine, Division of Endocrinology, Radboud University Nijmegen Medical Center, Nijmegen, Netherlands

**Keywords:** CSCs, embryogenesis, miRNAs, PGCs, PSCs, retrotransposons, tumorigenesis

## Abstract

Cancer is conventionally viewed as a disease of accumulated somatic mutation and epigenetic dysregulation leading to cell de-differentiation and uncontrolled proliferation. With few exceptions, malignant tumors develop from a single damaged cell. However, there is also strong evidence for the involvement of more than one cell in the initiation of oncogenesis. Oncogenic mutations may be insufficient by themselves to trigger oncogenesis as somatic cells harboring driver mutations are often seen in nonmalignant tissues. We review experimental evidence for the reactivation of embryonic genes, emergence of cancer stem cells, and therapy resistance by the developmental programme hijack via cell fusion. Furthermore, based on our previous findings on fetal-maternal microchimerism, in this hypothesis article we delve into the potential mechanisms of activation of the early totipotent program by unselective stem cell fusion for cell rescue, centering the primitive pluripotent stem cells residing in postnatal human tissues as potential pivotal drivers of tumorigenesis that could recapitulate incomplete stages of embryogenesis and cell migration after triggering nuclear reprogramming toward a totipotent zygote-like cancer stem cell state, potentially amenable to genomic instability, somatic mutation, defective histogenesis and tumor-host microchimerism.

## 1 Introduction

Preimplantation, implantation, growth, immunologic acceptance and fetal-maternal microchimerism are embryonic developmental processes that resemble the current hallmarks of cancer in many aspects like aerobic glycolysis/Warburg effect, phenotypic plasticity, cell invasiveness and migration, induction of neoangiogenesis, meiotic gene expression, epigenetic regulation, retrotransposon activity, protein profiling, immune escape, and proliferative signaling (Ma et al., 2010; Hanahan, 2022; Krisher and Prather, 2012; Geck, 2013). However, in spite of their resemblance, some hallmarks of cancer such as genome instability, mutation and metastasis are not hallmarks of embryogenesis. Still, this suggests that the shared mechanisms of the two processes might involve recapitulation of embryonic developmental programmes at least up to a point that could potentially be evoked by a similar type of trigger such as cell-cell fusion in both occurrences.

The last decades have brought about major advances in understanding cell-cell fusion. Hybrid cells resulting from fusion between gametes, myoblasts, epithelial cells, immune cells, trophoblasts, cancer, and other cells in physiological and pathological processes have been extensively explored (Sapir et al., 2008; Posey et al., 2011) (Table 1). Specific cell fusion processes were shown to proceed through related membrane rearrangements. Fusogens, the proteins required for cell fusion on either one of both fusing cells use varying mechanisms as some fusions are controlled by a single fusogen, while others depend on several proteins that either cooperate throughout the fusion pathway or are the drivers of distinct fusion stages (Sapir et al., 2008). Nonetheless, some fusions require fusogens to be present on both fusing membranes, while in other contexts, fusogens are required only on one of the membranes. How and why cells fuse in both normal development and disease still remains an active area of research but important progresses have been made to explore this process by both *in vitro* and *in vivo* assays (Brukman et al., 2019).

**TABLE 1:** Various types of cell fusion and their resulting entities in physiological, reparatory and cancer related conditions.

Mechanism	Type of fusion	Cell #1	Cell #2	Resulting entity	References
Physiological	Heterotypic	Egg cell	Sperm cell	Zygote	Bianchi et al. (2014)
	Homotypic	Mioblast	Mioblast	Syncytial multinucleated myofibers	Posey et al. (2011)
		Cytotrophoblast	Cytotrophoblast	Syncytiotrophoblast	Pötgens et al. (2004)
		Osteoclast precursor	Osteoclast precursor	Multinucleated osteoclast	Levaot et al. (2015)
Reparatory	Heterotypic	Neural stem cell	Neuron	Hybrid neuron	Cusulin et al. (2012)
		Bone marrow stem cell	Damaged hepatocyte	Hybrid functional hepatocyte	Vassilopoulos et al. (2003)
		Mesenchymal stem cell	Damaged epithelial intestinal cell	Hybrid functional epithelial cell	Ferrand et al. (2011)
		Mesenchymal stem cell	Degenerated Purkinje neuron	Hybrid functional neuron	Bae et al. (2007)
		Bone marrow stem cell	Damaged cardiomyocyte	Hybrid functional cardiomyocyte	Bittner et al. (1999)
Cancer related	Heterotypic	Cancer cell	Macrophage	Hybrid metastatic cancer cell	Pawelek (2000)
		Cancer cell	Bone marrow stem cell	Highly metastatic cancer cell hybrid	Kerbel et al. (1983)
		Cancer cell	Somatic cell	Differentiated cell hybrid	Halaban et al. (1980)
		Cancer cell	Cancer cell	Highly metastatic cancer cell hybrid	Miller et al. (1988)
	Homotypic	Hodgkin cell	Hodgkin cell	Reed-Sternberg cell	Rengstl et al. (2013)

The hybrid cells resulting from fusion retain genetic and functional characteristics of the fusing cells defining a “cell chimera” in which the genome of the less differentiated cell is dominant over the more differentiated cell, likely due to expression of key reprogrammers. The “cell chimera” can result from homotypic cell fusion when the fusing cells are identical, while heterotypic fusion occurs when cells of different origins of the organism fuse together forming a heterokaryon (stable di-nucleated hybrid cell) or synkarion (hybrid cell with a single nucleus but double in the genetic material) (Lluis and Cosma, 2010). This type of “cellular chimerism” is different from the classical intercellular chimerism with primitive pluripotent fetal stem cells occurring early in pregnancy during gastrulation (fetal-maternal microchimerism) or with bone marrow derived stem cell transplantation (donor cell chimerism) in which fetal or donor cells home and coexist with host cells in various niche of the host tissues (Cismaru et al., 2018; Cismaru et al., 2019; Cismaru et al., 2020).

Starting from the evidence that primitive pluripotent stem cells (pPSCs) with germline traits reside in the quiescent state in postnatal tissues as epiblast derived vestiges from development (e.g., very small embryonic-like stem cells – VSELs) (Ratajczak et al., 2019) and that fusion between stem and differentiated cells harboring mutations can occur unselectively for cell rescue (Blau, 2002; Lluis and Cosma, 2010), the current analysis posits a hypothesis that, although all cancers originate from a single cell, the tumorigenesis process initially involves two types of cells, one pluripotent with germ line traits and one somatic but damaged by environmental insults which fuse together for cell rescue. It is due to the intrinsic ability of germline related pluripotent cells to fuse with somatic cells and reprogram the somatic genome using Tet1/Tet2 for imprint erasure (Piccolo et al., 2013) that a potential result of such fusion may actually constitute a second hit in the damaged cell leading to the generation of a zygote-like cancer stem cell (Carnegie stage 1 equivalent) via somatic cell nuclear reprogramming which subsequently drives a tumor development that recapitulates the initial Carnegie stages of embryogenesis but with a high rate of developmental abnormalities caused by mutation and epigenetic remnants in the damaged somatic cell altering gene expression regulation. The functional interrelation between germ cells which already possess critical reprogramming factors for development and somatic cell reprogramming (Hu et al., 2018) indicates that the (failed) rescue attempt by germline related cells using fusion-driven reprogramming is grounded in established molecular mechanisms.

In the following paragraphs we bridged a wide array of complex, multidisciplinary concepts that explore physiological and pathological cell fusion, germline traits, homing mechanisms, miRNA regulation, and retrotransposon activation, supporting the current germ-somatic fusion hypothesis of ZL-CSCs emergence.

## 2 Physiological cell fusion

### 2.1 Stem cell fusion in organogenesis

While multiple physiological processes involve cell fusion, the prototype of selective cell fusion is best described in gamete fusion which occurs naturally in egg fertilization by sperm cells in humans. This further triggers the next stages of embryogenesis from cleavage, morulation, blastulation, implantation, gastrulation, neurulation, which involve host interactions that permit invasion of the miometrium, angiogenesis, cell proliferation, cell migration inside the embryo and at distant maternal seeding sites (e.g., fetal-maternal microchimerism), immunological modulation, reactivity restriction, and release of trophic hormones by the trophoblast (e.g., chorionic gonadotropin) leading to the development and growth of the fetus and its acceptance by the mother’s organism (Handschuh et al., 2007). These stages, regulated by demethylation of imprinted genes in the primordial stem cells of the blastocyst produce changes in the host tissues which provide nutritional support by host blood supply and immunological acceptance for the developing organism (Rossant and Tam, 2022; Ruvinsky, 1999). Syncytium formation by multiple cell fusions of uninuclear cells also occurs naturally in the decidualization process during embryo implantation (Wang and Dey, 2006).

A spontaneous cell fusion into syncytia also occurs naturally in muscle fibers during myogenesis. This interplay ensures the growth, maintenance and repair of muscle fibers throughout the life of an individual (Lehka and Rędowicz, 2020).

### 2.2 Stem cell fusion in tissue repair

Cells use an intrinsic DNA damage response network of repair mechanisms and cell cycle checkpoints for dealing with genomic insults caused by replication errors, environmental agents, ionizing radiation and genotoxic chemicals (Giglia-Mari et al., 2011). If DNA cannot be restored, the damaged cell transits to a senescent state and signals distress through senescence-associated secretory phenotype (SASP) inflammatory cytokines to initiate their clearance by immune cells and recruit progenitor cells to repopulate the tissue (Muñoz-Espín and Serrano, 2014; Wang et al., 2024). CXCL12/CXCR4 chemokine axis signaling via neighboring pericytic mesenchymal progenitors is pivotal for the recruitment of stem cells for tissue repair at the site of cell damage (Cismaru et al., 2022). Stem cells play important roles in tissue repair and regeneration, by mechanisms which range from replacement of senescent cells through replication and differentiation, to secretion of trophic biomolecules, and even transfer of mitochondria, an evolutionary conserved phenomenon of mesenchymal stem cells (MSCs) to reestablish mitochondrial function in cells harboring mitochondrial dysfunctions (Gomzikova et al., 2021). Accumulating evidence supports the notion of a new type of cell rescue by stem cell fusion which supplies a mutation-harboring cell with new genes (e.g., tumour-suppressor genes in cancer cells), or correcting, up to a point, damaged cells harboring genetic defects continuously throughout life (Blau, 2002; Brukman et al., 2019; Pesaresi et al., 2019). Bone marrow-derived MSCs have the ability to differentiate into a variety of cell types depending on their plasticity and are a potential source for epithelial tissue repair by fusion (Pesaresi et al., 2019). MSCs expressing pluripotency markers have been isolated from the bone marrow and other tissues suggesting origins in more primitive precursors (Cismaru et al., 2020; Ratajczak, 2015; Ratajczak et al., 2017). Several studies have demonstrated their ability to repopulate the gastrointestinal tract in bone marrow transplanted patients or in animal models of gastrointestinal carcinogenesis. However, mechanism of MSC epithelial differentiation still remains unclear and controversial with trans-differentiation or fusion events being evoked. In a study investigating the ability of MSCs to acquire epithelial characteristics, the authors showed that human bone marrow-derived stem cells (BMSCs) acquire epithelial characteristics through fusion with gastrointestinal epithelial cells (Ferrand et al., 2011). Neural stem cells derived from embryonic stem cells were shown to fuse with microglia and mature neurons both *in vitro* and *in vivo*, the hybrid cells retaining genetic and functional characteristics of both fused cells being able to differentiate into neurons and astrocytes (Cusulin et al., 2012). Dystrophic cardiomyocytes were shown to fuse with BMSCs for cell repair and not with normal myocytes in mice (Bittner et al., 1999), while neurons were shown to fuse with BMSCs in humans with hematologic malignancies and not healthy controls, both types of fusions resulting in intracellular chimerism. In another study, BMSCs fusion with mutation-harboring hepatocytes after BM stem cell transplantation (BMSCT) was also shown to regenerate liver by generation of hybrid cells containing both donor and host genes, consistent with polyploid genome formation by fusion of host and donor cells in the liver (Vassilopoulos et al., 2003). While polyploidy reduction by mitosis may lead to missegregation and cell abnormalities in daughter cells (Matsumoto et al., 2021), it was proposed that mutation-harboring cell rescue by fusion with stem cells in this “cloning-like” rescue mechanism may function as a genomic restorative process for highly specialized cells that rarely (if ever) replicate and are not amenable for replacement by new non-functionalized cells in organs such as brain, muscle or liver (Blau, 2002).

### 2.3 Artificial/induced cell fusion

Cell fusion for specific reprogramming purposes has been explored for more than a half of century in the technique of generating antibodies of predefined specificity and has been used for the production of monoclonal antibodies (Köhler and Milstein, 1975).

In recent years, the fusion with stem cells has been used to induce cell reprogramming. It was shown that pluripotent stem cells (PSCs), when fused with somatic cells, have the dominant capability to reprogram the somatic genome leading to somatic cell nuclear reprogramming (Ficz and Reik, 2013). Fusion allows the somatic cell to acquire homologous characteristics by genomic reprogramming and to dedifferentiate into PSCs (Piccolo et al., 2013).

Artificial laser-induced fusion between single hepatocellular cancer cells (HepG2) and human embryonic stem cells (hESCs) allowed for the generation of cancer stem cells (CSCs). CSCs represent a distinct subpopulation of neoplastic cells that have the capacity to promote tumor growth, support self-renewal, resist conventional therapies and drive metastasis and relapse. The occurrence of CSCs in solid tumors of a wide range of organs is firmly supported by the accumulating evidence in recent years (Clarke et al., 2006; Dittmar et al., 2009; Chen et al., 2013). With the newly acquired cancer- and stem cell - like characteristics, the resulting pluripotent hybrid cells resulted by fusion express increased tumorigenicity and drug resistance (Wang R. et al., 2016).

## 3 Pathological and cancer related cell fusion

Syncytium formation can be induced by specific types of viral infections, the most extensively described being the respiratory syncytial virus while cell-cell fusion is also documented in human immunodeficiency virus and herpes simplex virus leading to syncytium formation and enabling the viral genome transfer into neighboring cells (Leroy et al., 2020). Syncytium formation by fused pneumocytes was also observed in the severe stages of SARS-CoV-2 infection by pathology reports (Lin et al., 2021).

The hallmark cell of Hodgkin’s lymphoma is represented by Hodgkin and Reed/Sternberg (HRS) cell that does not resemble any normal cell in the body. These are large, often multinucleated cells with a dystrophic morphology and an uncharacteristic immunophenotype (Küppers and Hansmann, 2005). The HRS cell, usually derived from B lymphocytes, was shown to be the result of fusion events. Until recently, the classical notion through which HRS cells developed from mononucleated Hodgkin cells was thought to be the mechanism of endomitosis. However, using continuous single-cell tracking of Hodgkin lymphoma cell lines by long-term time-lapse microscopy, it has been established that cell fusion represents the primary mechanism of HRS cell generation (Rengstl et al., 2013). The implications of small mononuclear cell fusion of cells with different ancestors in the proliferative compartment of the Hodgkin lymphoma tumor clone goes beyond Hodgkin disease since HRS-like cells are frequently seen in various other diseases such as infectious mononucleosis, some Non-Hodgkin Lymphomas (e.g., T-cell lymphomas, B-cell chronic lymphocytic leukemia/small lymphocytic lymphoma - CLL/SLL), and even in EBV-associated lymphoproliferative disorders (Küppers and Hansmann, 2005). As HRS cell proliferation alone is insufficient for the expansion or maintenance of the HRS clone, it is arguable whether HRS cells have a pivotal role in pathogenesis or are simple reliques. While HRS cell generation implies cell membrane fusion of Hodgkin daughter cells or cousin cells which results in long-lived multinucleated low-proliferative giant cells, only small Hodgkin cells can maintain the HRS cell clone in culture. This is in contrast with the other population of growth-induced HRS cells which become polyploid through incompletely elucidated mechanisms and remain quiescent (Rengstl et al., 2013).

Stem cell fusion can lead to tumor heterogeneity through diverse genetic alterations. This heterogeneity can lead to the emergence of subpopulations of cells with distinct phenotypes contributing to the clonal evolution and adaptation of tumors, making them more challenging to target effectively (Marusyk and Polyak, 2010). Ploidy abnormalities in malignant tumors are common occurrences and are now considered a hallmark of cancer. Polyploidy and whole genome doubling has been documented in about 30%–37% of malignant tumors using whole genome sequencing (Bielski et al., 2018; Zack et al., 2013). While the mechanisms of polyploid cell generation in premalignant and malignant cells is still elusive, it has been argued that the extra set of chromosomes is not redundant and their genome may play a tumor suppressor role limiting tumor transformation by providing an additional set of tumor suppressor genes as a repair mechanism (Zhang et al., 2018; Lin et al., 2020). However, polyploidy reduction and missegregation following mitosis in replicating cells may potentially represent an early step in the initiation of oncogenesis (Matsumoto et al., 2021). As aneuploidy is observed in more than 90% of all solid tumors (Williams and Amon, 2009), this raises questions on how the aberrant caryotype may be part of tumor suppression or tumor progression mechanisms and what could be the actual implications of cell-cell fusion events in these processes.

Reprogramming specialized cells to a pluripotent state outside the physiological conditions of normal fecundation can be achieved artificially through a) induced pluripotency using transfection of pluripotency transcription factors (Yamanaka factors), b) fusion of somatic cells with embryonic stem cells or embryonic germ cells, and c) somatic cell nuclear transfer (cloning) (Yamanaka and Blau, 2010). Zygote formation by nuclear transfer technique from a somatic cell into an enucleated oocyte creates a clone with the donor cell’s genome (Tian et al., 2003). Non-enucleated oocytes can also be used for cloning leading to the disappearance of the oocyte DNA, but developmental abnormalities occur, caused by epigenetic remnants that alter gene expression regulation (Rouillon et al., 2019).

Activation of the totipotent embryonic genome requires accessing the higher-order chromatin structure by transient epigenetic programs which are specific to the zygote development of 2cell/4cell transcription state in which DUX4, ZSCAN4, TCSTV, and HERVL retroelement network play key roles (Hendrickson et al., 2017; Zhou and Dean, 2014; Defossez et al., 2022). This is only achieved during normal fecundation since germ cells are the only cells that undergo reprogramming to totipotency to the zygote state under physiological conditions. However, they also give rise to induced pluripotent stem cells (iPSCs) under the appropriate conditions *in vitro* since they already possess critical reprogramming factors. Despite that primordial germ cells (PGCs) are committed to produce unipotent cells, they express many of the master regulatory factors that facilitate pluripotency (Durcova-Hills et al., 2001). The germline specific gene Prmt5, which is pivotal for PGC development together with the other genes expressed in PGCs and involved in reprogramming such as OCT_3/4_, SOX_2_, and LIN28, have the potential to reprogram somatic cells into iPSCs *in vitro* that exhibit germline transmission (Nagamatsu et al., 2011). This indicates that there is a functional interrelation between germ cell development and somatic cell reprogramming (Hu et al., 2018). Induced from PGCs, embryonic germ cells (EGCs) harbor an epigenome resembling to that of PGCs (Nagamatsu et al., 2013). Following fusion with somatic cells, EGCs have a key advantage in reprogramming the somatic genome over embryonic stem cells (ESCs). Unlike ESCs which maintain DNA methylation in their imprinted control regions (ICRs), EGCs possess erased DNA methylation in ICRs and upon fusion with the somatic cell, erasure of DNA methylation in the ICRs of the somatic genome occurs which reprograms the somatic cell to a zygote-like state (Ficz and Reik, 2013). This cell-fusion mediated imprint erasure and reprogramming in the somatic genome is modulated by Ten-Eleven Translocation proteins Tet1 and Tet2 leading to accumulation of 5-hydroxymethylcytosine (5hmC) and DNA demethylation at several ICRs following fusion with EGCs (Piccolo et al., 2013).

*In vivo,* the closest postnatal equivalent of an EGC is an early germ cell (Zwaka and Thomson, 2005). Persistent missmigrated/ectopic PGCs that fail to enter apoptosis and retain pluripotency, have been sown to reside in postnatal tissues being amenable to tumorigenesis later in life (Stallock et al., 2003; Heaney et al., 2012). Whether fusion events become involved in the missmigrated PGC related tumorigenesis still remains elusive but essential aspects such as the generation of multiple phenotypic tumor sub-clones that contribute to intratumoral heterogeneity, transition between epithelial, mesenchymal, and stem-like states, high capacity for tumor initiation *in vivo*, strong resilience to environmental stress and resistance to therapy linked to dormancy and reprogramming pathways could potentially be achieved *in vivo* by a germ-somatic fusion event connecting oncofetal and embryonic reprogramming pathways as further discussed in the next section.

### 3.1 Cell-cell fusion in oncogenesis

Stem cells have traits that entail them with high survival capabilities under unfavorable environments which include (I) quiescence, (II) active DNA-repair mechanism, (III) efflux transporters for toxic agents, (IV) high detoxification metabolism, and (V) apoptosis reluctance. Such mechanisms are believed to also be employed by CSC, to evade anti-cancer drugs (Ng et al., 2013). CSCs have pivotal roles in tumorigenicity, drug resistance as well as recurrence (Beck and Blanpain, 2013). Among cancer cells, CSCs were shown to possess several traits, such as a slower cycling or dormancy in some contexts, anti-apoptosis, downregulation of anti-proliferative pathways, drug resistance and more efficient DNA damage repair capabilities, which make them the protagonists of tumor drug resistance and recurrence (Batlle and Clevers, 2017). The American Association of Cancer Research (AACR) defined in 2006 a CSC as any cancer cell that possessed stem cell-like properties of multi/pluripotency and unlimited self-renewal and specifically highlighted that the definition of a CSC does not imply that is has to be that initial cell in the body that caused cancer or its provenience from the tissue resident stem cells affected by tumor transformation (Clarke et al., 2006). The widely accepted dogma of CSCs occurrence is that CSCs may be derived from normal stem cells, which may be affected by cancer causing somatic cell mutations (Takebe et al., 2015; Trosko, 2021). However, as recurrent tumors often exhibit different characteristics and phenotypes compared with the original tumors, another hypothesis about the origin of CSCs is that CSCs are derived from the fusion of stem cells and differentiated cells (Dittmar et al., 2009). With the presence of genomic hybrids and polyploid cells in both premalignant and malignant tissues, fusion events have gained a special interest as hallmarks of oncogenesis (Spandidos Publications, 2025a).

The exact moment and mechanism by which a somatic cell or a stem cell becomes a CSC is not completely understood. As it may take years for mutations to develop and accumulate in a somatic cell, a germline mutation is acquired upon fecundation and represents the starting point of cancer susceptibility syndromes which can cause cancer in newborns and children (Pruteanu et al., 2020; Sarkozy et al., 2009; Carvalho et al., 2021). However, how a mutated cell acquires or rejects tumorigenicity is still unknown but cannot exclude the involvement of stem cell fusion events in this process. This is since various oncogenic driver mutations have been observed in both nonmalignant and malignant lesions. Selected examples include BRAF mutation, an activating mutation in about 50% of melanoma cases, also present in more than 50% of benign nevi and atypical melanocytic nevi (Uribe et al., 2003). ALK and EGFR activating mutations in non-small cell lung cancer (NSCLC) are common occurrences in benign lung lesions (Hyde et al., 2023; Kamata et al., 2016) while PTEN activating mutation in endometrial cancer is also reported in endometriosis–a nonmalignant pathology involving ectopic endometrial tissue (Govatati et al., 2014) (Spandidos-Publications, 2025b). The presence of IDH–mutant progenitor cells in non–cancerous regions of the brain in glioblastoma patients indicates that it is subsequent events that may or may not lead to the tumor transformation of IDH-mutation harboring glial and oligodendrocyte progenitor cells migrated during embryogenesis (Park et al., 2026). As oncogenic driver mutations in cancer are also seen in benign lesions, it is arguable whether these mutations are sufficient by themselves for malignant transformation, or whether subsequent events such as maybe fusion for cell rescue could potentially trigger genomic reprogramming and oncogenesis at some point during the development of the lesion. Such potential sequence of unfavorable events seems to be contrary to the main objective of repair mechanisms by fusion so another pivotal trigger could drive the oncogenic process. It becomes intriguing to speculate that the ontogeny of unanticipated but versatile stem cells involved in cell rescue by fusion could drive a twist of fate in the resulting hybrid (Cismaru et al., 2022).

## 4 Germline traits in tumors: on the potential origin of the zygote-like-CSC

Unlike CSCs which have been described as pluripotent and lineage restricted, zygote-like (8C-like) CSCs have been described as a rare metastable subpopulation of 1 in 1,000 cancer DUX4^+^ HLA-I^-^ cells that are able to undergo ZGA and go through an early totipotent program with the expression of embryonic, trophectoderm, and mesenchymal markers (Smith et al., 2023). Since DUX4 overexpression in humans is characteristic of the 2-4-cell stage zygote (Hendrickson et al., 2017) and also of early germ cells (Karlsson et al., 2021), they sit conceptually between cancer stem cells (CSCs) Induced pluripotent stem (iPS)-like states and pre-implantation embryonic cell states. We will further refer to these CSCs as zygote-like (ZL-CSCs) as they can be defined as a rare, highly plastic tumor cell population that expresses totipotency-associated genes and epigenetic features similar to those found in the zygote, enabling the cell to self-renew and differentiate into multiple divergent tumor cell lineages, thereby driving tumor initiation, progression, immune escape, and therapy resistance.

In order to better understand the putative origin of ZL-CSCs, we delved deeper in ontogeny, to primitive progenitors emerging during gastrulation which have been shown to migrate and colonize fetal and maternal tissues, becoming a source of steady-state tissue committed pluripotent stem cells with a tight germ line kinship and overlapping molecular and functional traits with primordial germ cells (PGCs), primitive HSCs, primitive MSCs, and other pluripotent tissue resident precursors that have received different names depending on the teams that identified and described them (Howell et al., 2003; Ratajczak et al., 2017) but most probably indicate similar or overlapping primitive populations of pluripotent cells (Ratajczak et al., 2012; Cismaru et al., 2019; Cismaru et al., 2020). We will further address these cells as primitive pluripotent stem cells (pPSCs) since a constant feature indicating there primitiveness and linking them to other populations of PSCs is their induction in numbers in response to stimulating a vestige from their ontogeny – the orphan LHCG receptor by pituitary hormones, sex hormones, and chorionic gonadotropin (Mierzejewska et al., 2015; Cismaru et al., 2020; Ratajczak, 2017; Abdelbaset-Ismail et al., 2015). Another characteristic of pPSCs in postnatal tissues is their susceptibility to tumorigenesis by still incompletely elucidated mechanisms which range from oncogenic transformation into CSCs and genomic instability following exposure to environmental insults (Bhartiya, 2025), to fusion with somatic cells leading to heterokaryon formation, chromosomal aneuploidy and genomic instability leading to malignant transformation (Ratajczak et al., 2009; Ratajczak et al., 2017). Having germ line markers, these pluripotent but lineage restricted stem cells are far more advanced in their differentiation pathway than a totipotent zygote, but are the closest in kinship with germ cells that acquire totipotency through fertilization (Ratajczak, 2017). Our hypothesis for the potential origin of ZL-CSCs is that they could potentially emerge from the unselective but fecundation-like fusion of tissue resident pPCS harboring germline traits, with mutation harboring somatic cells, for cell rescue.

The most versatile germ line cells are the PGCs which during development migrate by a well-defined route from the yolk sac into the genital ridge later giving rise to gametes throughout life (Inst, 1948). They are the only cells in the human body capable of both mitosis and meiosis. During mitosis, they maintain their diploid state. During meiosis, the ploidy is reduced from diploid (2n) to haploid (1n) in the development of eggs and sperm cells. PGCs are programmed to go into apoptosis by BAX mediated programmed cell death if they don’t reach their destination (Stallock et al., 2003), but some PGCs may mismigrate and hijack apoptosis later in life giving rise to germ cell tumors across their continued perineural migration along the midline of the developing embryo in distant sites of the sacral region, retroperitoneum, abdomen, anterior mediastinum, neck and midline of the brain as proposed by the embryonic rest hypotheses of Rudolf Virchow and Julius Cohnheim (19th century) (Oosterhuis and Looijenga, 2019; Ratajczak et al., 2013). The striking resemblances between PGC-like cancer cells and PGCs in cell features like gene expression profiles such as CXCR4 and c-Kit, migration and tumor metastasis potential have been previously explored extensively (Liu C. et al., 2022). The germline traits seen in tumors which are further discussed in the next section represent indirect evidence for the potential involvement of PGC-related pPSCs from postnatal tissues in tumorigenesis.

Akin to the PGCs, many cancer cells express genes linked to different embryo developmental stages, such as germ cell-specific genes. This allows them to express meiotic proteins and undergo meiosis similar to PGCs (McFarlane and Wakeman, 2017). Two genes regulating pluripotency in embryonic development that greatly influence meiosis in both PGCs and cancer cells are LIN28A and LIN28B also known as germ-cell/cancer (GC) genes (Irie et al., 2015; Bruggeman et al., 2018).

Germline-specific genes are often activated in cancer. Genes specific to PGCs, such as the germ cell determination gene DAZL, modulate tumorigenicity, stemness and drug resistance in cancer cells, being associated with the expression of stemness markers such as PROM1, SOX2, NANOG in glioblastoma cells (Zhang et al., 2020b), while in NSCLC it promotes migration, invasion and drug resistance by upregulating JAK2 and MCM8 (Zhou et al., 2024). NANOS3, another germline specific gene that regulates inhibition of apoptosis during migration of PGCs, is also expressed in glioblastoma where it promotes migration, proliferation, invasion and drug resistance (Zhang et al., 2020a) while in lung cancer it promotes invasiveness by inducing EMT (Grelet et al., 2015).

DUX4 gene is an important regulator of ZGA for totipotency in humans and its silencing leads to disordered degradation of maternal transcripts and incomplete ZGA (Liu Y. et al., 2022). While DUX4 is repressed in somatic cells, it is expressed in high levels in germ line cells (Snider et al., 2010). A single–cell type transcriptomics map of human tissues and subcellular map of human proteome shows an increased expression in early germ cells but many cancer types also show a high expression of DUX4 RNA (Karlsson et al., 2021; Thul et al., 2017). In cancer cell lines, DUX4 is only expressed in a subpopulation of 0.01% of cells under standard culturing conditions, but its transient (zygote-like) expression is sufficient to induce early embryonic and extraembryonic totipotent programs with expression of genes regulating trophoectoderm formation, and selective immune evasion by suppression of MHC class I (Smith et al., 2023; Chew et al., 2019).

Trophoblast specific genes such as TACSTD2 encoding Trop-2 expression are specific to trophoblast cells and developing embryo tissues warranting them invasiveness (e.g., invasion of the myometrium and induction of neoangiogenesis for access to host blood supply) and immune tolerance. However, Trop-2 overexpression is seen in many tumor types being associated with increased aggressiveness (Shvartsur and Bonavida, 2015). Trophoblast cells also express HLA-G, a non-classical HLA Class I antigen involved in the induction of immunological acceptance of the embryo by the host tissues of the mother and recognized as a hallmark of the placenta (Loke et al., 1994). HLA-G is strongly linked to embryogenesis being expressed in the zygote, blastocyst and early embryo, and its roles in spermatogenesis further links its expression to PGCs (Yao et al., 2014). Our previous findings *in vitro* show that HLA-G expression is associated with the selection of more primitive and potent progenitors following human chorionic gonadotropin (HCG) exposure in primary cultures of BM derived stem cells (Cismaru et al., 2020). In cancer, HLA-G expression is a common occurrence endowing cancer cells with immune evasion and metastatic capabilities (Lin and Yan, 2015). Syncytins, the only known human fusogens, encoded by ERVW-1 gene, are proteins expressed by cytotrophoblast and syncytiotrophoblast cells of the feto-placental barrier that allows nutrient and waste exchange but blocks maternal immune cell infiltration (Sha et al., 2000). Remarkably, syncytyn-1 is also expressed in microglia (Antony et al., 2004), and testis (Sha et al., 2000), potentially linking its expression to yolk sack progenitors such as primitive HSCs and PGCs since microglia is known to originate from the yolk sac progenitors and colonize the brain before the establishment of the blood-brain barrier during embryogenesis (Dermitzakis et al., 2023). ERVW-1 gene and Syncytins expression is also upregulated in early germ cells and its expression is superposable to NANOS3 gene expression in germ cells and cancer cells (Uhlén et al., 2015). It is noteworthy that various cancer cells express syncytyn-1 endowing them with fusion capabilities in breast cancer (Bjerregaard et al., 2006), endometrial cancer, testicular cancer/seminoma, lung cancer/NSCLC, prostate cancer, colorectal cancer, bladder cancer, neuroblastoma and cutaneous T-cell lymphoma/mycosis fungoides (Wang et al., 2023). Furthermore, IZUMO1 gene, encoding the Izumo sperm-oocyte fusion protein in sperm cells together with its receptor in egg cells (JUNO/folate-receptor delta isoform/SRPδ) is involved in gamete binding during fecundation and in meiosis (Bianchi et al., 2014). IZUMO1 has been reported as a likely causal gene in various tumors such as melanoma, head and neck squamous cell carcinoma (Iorio et al., 2018), colorectal cancer (Law et al., 2019) and leukemia (Halik et al., 2020).

This link between germ line cells and cancer stem cells, underlined by the cancer/testis antigens encoded by genes with restricted expression to the germline, but also expressed in a range of cancers, has been extensively explored in the last years (Bruggeman et al., 2018). The recapitulation of parts of the germline gene expression programme (gametic recapitulation) is reflected in oncogenesis through immortalization, invasiveness, immune escape, hypomethylation and migration/metastatic spread, underscoring the shared characteristics of germ cells and cancer cells (Simpson et al., 2005). A comparison between germ cell transcriptome and combined data from public cancer genome databases (TCGA, GTEx) showed more than 700 genes that are highly germ cell-yet also cancer-specific, being essential for germ cell development, but also for tumor proliferation and metastasis (Bruggeman et al., 2018; Wang C. et al., 2016).

**FIGURE 1:**
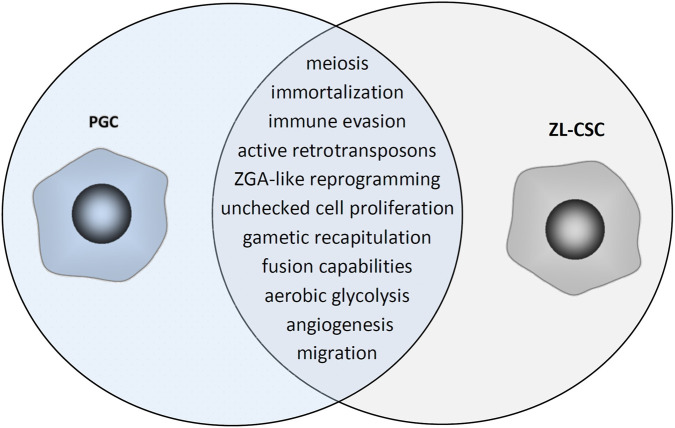
Venn diagram displaying the overlapping traits shared by primordial germ cells (PGCs) and zygote-like cancer stem cells (ZL-CSCs) from the germline gene expression programme. Persistent and quiescent missmigrated/ectopic PGCs that fail to enter apoptosis and retain pluripotency are the most primitive PSCs from postnatal tissues and are presumably part of similar populations of developmentally related (epiblast derived) tissue resident quiescent pPSCs involved in tumorigenesis since their plasticity is well reported into all 3 germ layers and into germ cells.

While expression of meiotic genes is a common trait in both germ and cancer cells, the mechanisms leading to such expression in CSCs are still elusive, with dedifferentiation potentially being evoked (Lingg et al., 2022; Sou et al., 2023). However, given the striking similarities between PGCs and CSCs both genotypicaly and phenotypicaly (Figure 1), fusion events could bring more insights into how a mutation-harboring cell could acquire meiotic genes and become a ZL-CSC. This is since erasure of the gametic imprinting of genes in the zygote or the primordial cells of the blastocyst and the totipotent stem cells of the very early embryo, control not only the fetal growth but also the feto-maternal interactions that are required to maintain a balance between contradictory fetal and maternal requirements including immunologic acceptance (Ruvinsky, 1999). As erasure of the gametic imprinting is regarded as a hallmark of cancer (Rainier and Feinberg, 1994), it becomes more clear how the dysregulated control mechanisms governing cell cycle progression in cancer resemble the unchecked cell division seen during embryonic development (Vogelstein and Kinzler, 2004).

**FIGURE 2:**
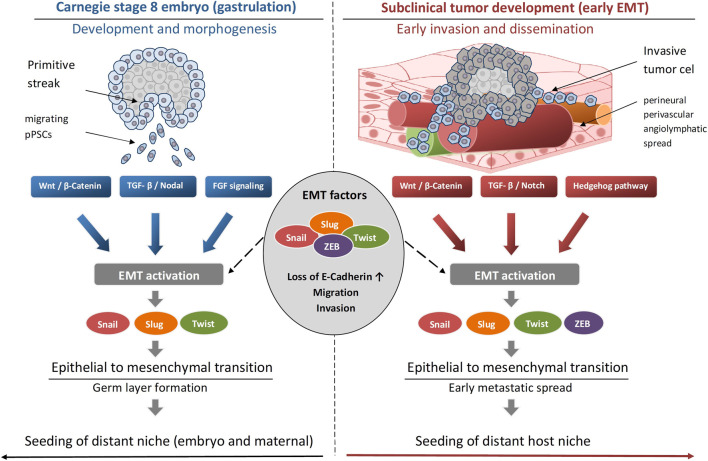
Graphical representation of the common traits shared by the developmental stage of a gastrula (Carnegie stage 8 embryo) and a subclinical stage of a tumor (tumoroid-like) in terms of the epithelial to mesenchymal transition (EMT) programme.

It is noteworthy that similarities between primitive germ line derived stem cells and CSCs exist not only in their genotype (Table 1) but also in their phenotype and are also reflected in the migratory capabilities in both circumstances (Figure 2). Exploring migratory events and homing of stem cells in predefined niche is an area of active research in both development and oncogenesis and is described in the following section.

In physiological conditions, anchorage dependence and contact inhibition of proliferation and migration are conventional mechanisms ensuring normal tissue homeostasis while a prolonged detachment usually triggers anoikis, a form of programmed cell death (McClatchey et al., 2016). In the physiological cell migration of PGCs, NANOS3 gene ensures the suppression of both Bax-dependent and -independent apoptotic pathways, being essential in the early stage embryo to protect the migrating PGCs from apoptosis. However, in PGCs that fail to reach their destination in the prospective gonads, the pro-apoptotic gene Bax is required for the death of ectopic PGCs (Stallock et al., 2003). Deregulated Bax expression is seen in ectopic germ cells that retain their primitive markers and are associated with germ cell tumors (Cook et al., 2011). Expression of pro-apoptotic gene BAX is regulated by the tumor suppressor p53 but about 50% of all tumors have a disrupted P53 function (Lomax et al., 1998).

In embryogenesis, the collective migratory events of gastrulation and neural crest cell development have been extensively described and imply clusters of primordial cells which migrate and become specified at locations distant to where they will ultimately reside for their physiological function. Collective epithelial and mesenchymal cell migration during gastrulation is based on mechanisms of epithelial to mesenchymal transition (Chuai et al., 2012) (Figure 2).

Seeding of primitive migratory cells in their prospected niche occurs physiologically during fetal development with the best examples being the migration of PGCs from the yolk sac to the genital ridge as future gametes, the migration of the neural crest cells to their destination in the nervous system, skin, and hair bulge as neurons/glia and melanocytes, or the migration of hemangioblasts from the yolk sac mesoderm to the primary vascular plexuses as angioblasts and of primitive HSCs to the aorta-gonad-mesonephros (AGM) then fetal liver and bone marrow (Møllgård et al., 2010; Kim, 2010). It has been reported that most tissues in the developed organism accommodate niche containing clones of primitive HSCs derived from the yolk sac that are capable of reenacting extramedullary hematopoiesis in organs other than the bone marrow such as lymphnodes, liver, spleen, adrenal glands, cranial and spinal nerves, prostate, pleura, peritoneum, and other tissues (Kim, 2010; Sohawon et al., 2012). Expression of CXCL12 (SDF1) is important for primitive HSC migration, homing, retention in the steady state and hematopoiesis in both the bone marrow and extramedullary sites (Wang et al., 2014; Schyrr et al., 2024; Mendt and Cardier, 2015). Furthermore, CXCL12a protein, the most widespread splicing variant which is more potent in tissues than in the blood (Janowski, 2009) is necessary to also guide PGCs toward the prospective gonad and ectopic expression of CXCL12a is capable of trapping PGCs in islands of high expression (Aman and Piotrowski, 2010). It was shown that PGCs can be attracted to ectopic sources of CXCL12 expression in extragonadal tissues (Doitsidou et al., 2002) therefore the expression of CXCL12 can be regarded as an important regulator of the suitability of the niche for both PGCs and primitive HSCs.

Yolk sac derived primitive HSCs exist in many adult tissues where they undergo asymmetrical division to self renew and symmetrical division to differentiate and produce tissue-resident immune cells such as histiocytes of the brain (microglia), liver (Kupffer cells), bone (osteoclasts), skin (Langerhans cells), lung (interstitial macrophages/nerve- and airway associated macrophages), lymph node and spleen (interdigitating cells), pleura (pleural macrophages), peritoneum (peritoneal macrophages), synovial lining (aquaporin 1^+^ macrophages), etc. Their pools are maintained independently of BM monocytes having a more tolerogenic phenotype than their BM-derived counterparts (van Furth, 1998; Bartnicki et al., 2024; Schonfeldova et al., 2025). Their niche with high CXCL12 expression seems to represent an appealing lodge for precursor cells including cancer cells (Hashimoto et al., 2013; Cismaru et al., 2022; Casanova-Acebes et al., 2021). Reports showing increased CXCL12 expression in tissues that are amenable to accommodating both extramedullary hematopoiesis (Kim, 2010) and metastases of various tumor types (Zhao et al., 2011; Köhler and Milstein, 1975; Pan et al., 2006; Tang et al., 2019; Oonakahara et al., 2012) support the role of the hematopoietic niche in hosting migrating stem cells. Anticancer therapies focused on targeting CXCL12/CXCR4 axis hold considerable promise due to its role in promoting proliferation of tumour cells in protective niches (Rueda et al., 2025).

It has been postulated that tissue-resident HSCs of the adult have common ancestry with primitive precursors related to the germ line such as PGCs and other epiblast derived stem cells (Mierzejewska et al., 2015; Ratajczak and Suszynska, 2016; Ratajczak et al., 2017). Evidence supporting this ontogeny is their expression of the orphan receptor - luteinizing hormone chorionic gonadotropin (LHCGR) that is stimulated by the pregnancy hormone - human chorionic gonadotropin (HCG) as well as pituitary and gonadal hormones (TSH, FSH, LH, estrogens, androgens) and their independence of erythropoietin which is required for definitive hematopoiesis (Ratajczak, 2017; Abdelbaset-Ismail et al., 2015; Maggio et al., 2013). Both Notch 1 and erythropoietin (EPO) are regulators of hematopoiesis by HSCs while they are not required for hematopoiesis by primitive-HSCs (pHSCs) (Huyhn et al., 1995; Pereda Tapiol and Niimi, 2008; Maggio et al., 2013). These observations emphasize the long documented positive effects of pituitary and placental hormones on stimulating hematopoiesis in bone marrow insufficiency (Maggio et al., 2013), in normal pregnancy (Dockree et al., 2021), in the hematopoietic effect of thyroid hormones (Zhang et al., 2017) and even the rejuvenating effect of pregnancy on the mother and her post reproductive life expectancy (Falick Michaeli et al., 2015; Grundy and Tomassini, 2005). Our group showed that HCG stimulates *in vitro* the selection of primitive precursors (CD133^+^, NANOG^+^, SOX_2_^+^, OCT_4_^+^) with a molecular profile linked to the gene expression programme of gastrulation (Cismaru et al., 2020), while *in vivo* HCG stimulates the induction of primitive hematopoietic precursors (CD34^+^, SCA1^+^) and leukocyte progenitor cells (CD29^+^, CD11b^+^) which lead to improved hematopoiesis and better survival in a murine model after peripheral blood stem cell transplantation (Cismaru et al., 2024). Reports of high levels of fetal hemoglobin (HbF) in adults with increased levels of HCG from germ cell tumors (Dainiak and Hoffman, 1980) also indicate that is rather the pHSCs that induce in numbers from the effect of HCG. A puzzling observation is the secretion of HCG not only by germ-cell tumors but also by various other tumor types. Paracrine secretion of β-HCG by malignant tumors such as osteosarcomas, lung, bladder, breast and colorectal cancers is associated with a worse prognosis (Glass et al., 2015; Iles et al., 2010). From these observations we can speculate that it is the tissue resident primitive stem cells with a germline kinship that play a pivotal role in tumorigenesis through the effect of HCG on their proliferation and on the aggressiveness and therapy resistance of tumors as also proposed by Bhartiya (2025). The group of Ratajczak et al. developed an elegant series of evidence showing that primitive stem cells with a germline kinship can have multiple implications in physiological and pathological conditions such as tissue regeneration, restoration of gametogenesis, hematopoiesis and even cancer (Ratajczak et al., 2017; Ratajczak et al., 2013; Ratajczak et al., 2007; Ratajczak et al., 2019).

While metastasis is a hallmark of oncogenesis, it does not represent a hallmark of embryogenesis. However, similar to embryonic migratory events of cells during gastrulation (Carnegie 8 stage embryogenesis), tumor cells in circulating clusters retain epithelial gene expression while also displaying a hybrid epithelial-mesenchymal phenotype to complete proliferation and metastasis in the early subclinical stage of tumor development (Cheung and Ewald, 2016) (Figure 2). Rather expectedly, distant sites of tumor cell clusters have been found in the early stages of cancer patients, which is in line with the observations that initiation of metastasis occurs in the subclinical stages of tumorigenesis (Yang et al., 2019; Steinert et al., 2008).

It is notable that subclinical tumor stages (1–2 mm^3^) share common traits with Carnegie 8 stage embryogenesis in their development such as upregulation of mesenchymal gene programmes with reactivation of the same EMT factors — Snail/Slug, Twist, ZEB — to reduce cell-cell adhesion and enhance migratory potential (Micalizzi et al., 2010), gastrulation signals such as Wnt, TGF-β, Notch, Hedgehog allowing invasion and migration (Kalluri and Weinberg, 2009; Garg, 2017), E-cadherin downregulation to acquire invasive traits (Scheibner et al., 2021; Kim et al., 2014) and dynamic EMT-MET ensuring plasticity which is essential for adaptation to changing microenvironments (Huang et al., 2022; Pirlog et al., 2023) (Figure 2).

Invasive migration by individual and collective cell clusters involves proteolytic extracellular matrix (ECM) remodeling (Wolf et al., 2007). The conventional mechanism of tumor metastasis holds that tumor cell migration begins with a single tumor cell and progresses through a number of intricate procedures before arriving and surviving at distant tissues and organs. Moreover, it has been demonstrated that the collective tumor cell migration, similar to physiological cluster migration during gastrulation, is more resistant to clinical treatments and has a larger capacity for invasion than the individual tumor cell migration. This is supported by findings that stem-cell-related and proliferation-related genes are abundant in circulating tumor cell clusters, and embryonic pluripotency transcription factors such as OCT4, NANOG, SOX2, and SIN3A have hypomethylated binding sites (Gkountela et al., 2019). Apart from the classical hematological and lymphatic spread, the metastatic migration of cancer cells along nerve sheaths is known as the perineural spread (Maroldi et al., 2008) while the abluminal perivascular migration of cancer cells is known as angiotropism and pericytic mimicry (Lugassy et al., 2014), recreating mechanisms of primitive cell migration that are specific to early embryonic development.

## 5 MiRNA regulation and retrotransposons

### 5.1 Micro RNAs at the basis of life as the oldest regulators of cell fusion

Cell fusion regulation is complex and tightly controlled, with growing evidence pointing to microRNAs (miRNAs) as essential modulators of the process. Quite expectedly, miRNAs, small (∼22 nucleotides), non-coding RNAs that regulate gene expression post-transcriptionally are not only ubiquitous regulators of gene expression, but also among the epigenetic mechanisms with the longest evolutionary history in cell fusion since early multicellular evolution (Christodoulou et al., 2010). From tissue identity to muscle formation, placentation, immune function and cell repair, miRNAs play essential roles in the control of cell fusion across various biological systems ensuring cellular cooperation and individuality. In mioblast fusion, miR-1, miR-133, and miR-206 regulate the differentiation and fusion of myoblasts into multinucleated myotubes by targeting factors like Pax7, Hdac4, and Sox6 (Chen et al., 2006). In syncytiotrophoblast formation, miR-193b and miR-17∼92 cluster have been implicated in trophoblast fusion by targeting key repressors of syncytin-1, a retroviral envelope protein essential for placental fusion (Morales-Prieto et al., 2012). MiR-223 is an important regulator of cells of the hematopoietic lineage playing a pivotal role in macrophage and osteoclast fusion (Sugatani and Hruska, 2007). SiRNA knockdown of miR-223 significantly increases osteoclast fusion while macrophages treated with mir-223 mimic (Moore et al., 2016) or pre-miR-223 (Sugatani and Hruska, 2007) lose their ability to undergo fusion in experimental models. There is growing evidence that the miR-200 family, miR-34, and other factors are linked to fusion-like activities and the epithelial–mesenchymal transition (EMT) in cancer stem cells, while their normal expression inhibits nuclear reprogramming of cells toward less differentiated phenotypes (Shimono et al., 2009; Choi et al., 2011).

MiR-17∼92 cluster (also known as oncomir-1), part of the let-7 family miRNAs is essential in early embryogenesis for hematopoietic and various fetal tissue development, and deficient miR-17∼92 cluster is associated with postnatal fatality (Ventura et al., 2008). A comparison of miRNA expression in the mature oocyte, with zygote miRNAs, showed the same miRNA expression pattern in both cells, consistent with a maternal inheritance of these miRNAs from oocytes and not their transcription in the zygote itself (Tang et al., 2007). Being essential for embryonic development, its germline transmission make it a pivotal regulator of gene expression in embryonic stem cells. It is becoming increasingly clear that in fusion capable cells such as primordial germ cells, embryonic stem cells and cancer stem cells, MiR-17∼92 cluster functioning is integral to the balance between “stemness” and differentiation, employed during development but hijacked in malignancy (Cioffi et al., 2015).

Murine studies have shown that upon egg fecundation, a maternal to zygote transition (MZT) occurs, which starts with the gradual clearance of maternal Dicer and miRNAs stored in the cytoplasm of oocytes, which are crucial to sustain the first cleavages in the first steps of early development (Tang et al., 2007). MiRNAs act pleiotropically, blocking the translation of hundreds of maternal targets and miR-17∼92 cluster plays a pivotal role in this process. The transition to zygotic genome ensures that the maternal transcripts are actively degraded and replaced by zygotic products during zygotic genome activation (ZGA), a striking example of cellular reprogramming (Rosa and Brivanlou, 2017). A new synthesis of miRNAs begins with the two-cell stage and includes the expression of miR-290 to miR-295 as the first detectable embryonic miRNAs (Tang et al., 2007). It is noteworthy that miR-290 to miR-295 are also specifically expressed in embryonic stem cells (ESCs), being associated with maintenance of pluripotency (Houbaviy et al., 2003). ESCs have a distinctive cell cycle with a very short G1 phase and deficient G1/S check point, ensuring them a quicker entrance in the S phase, similar with cancer cells (Berthet and Kaldis, 2007). This mechanism of pluripotency maintenance through shortening G1 and extending S phase is assisted by miR-290–295 cluster through its effects on Cyclin D1 (Yuan et al., 2017). The increased expression of Cyclin D1 that drives unchecked cellular proliferation is regulated postranscriptionally by miR-290–295 cluster in ZGA and is also recapitulated in cancer, promoting tumor growth and drug rezistance (Montalto and De Amicis, 2020). The S phase regulating kinase CDK2 is not essential for mitotic cell cycle as it is compensated by CDK1 which promotes G1/S transition. However, in germ cells, CDK2 is essential for meiosis as it cannot be compensated by CDK1 (Berthet and Kaldis, 2007). It was shown that failure to downregulate Ciclin D1 in primordial germ cells in order to go on cell arrest on schedule is associated with germ cell tumors susceptibility (Lanza et al., 2016; Cook et al., 2011; Heaney et al., 2012). The expression of the miR-290–295 cluster in mice and its miR-371–373 homolog in humans is restricted to early embryos, stem cell lines derived from the early embryonic lineages, PGCs, and the germ line stem cell compartment of the adult testis (Wu et al., 2014), providing a link between the stemness potential of PGCs and CSCs (Figure 1).

Despite having established roles in regulating post-transcriptional gene expression after fusion of diverse cells, there aren’t any miRNAs reported in the existent literature that regulate the expression of fusion genes such as IZUMO1 and ERVW-1 which are essential in gamete adhesion and fusion (Soygur and Sati, 2016). As these genes evolved under strong transcriptional containment, with relatively minor selective pressure to develop complex miRNA-based repression layers, this suggests that regulation relies more on transcriptional silencing than on post-transcriptional modulation so their reactivation is thus mostly transcription-driven, not miRNA-limited. This supports the idea that, while serving as ancient epigenetic sentinels ensuring that post-fusion events occur only under tightly regulated conditions, miRNAs have fewer (if any - they are difficult to predict, rarely investigated, and biologically non-canonical) regulatory implications during the actual fusion. This is particularly important because dysregulated fusion may potentially lead to immunological dysfunction, defectuous histogenesis and even multinucleated or polyploid tumor cells (Shabo et al., 2020).

### 5.2 Transposable elements as key players in early embryogenesis and cancer

Epigenetics play a key role in development, determining cell fate by driving the transition from a single totipotent stem cell to an entire organism during embryogenesis. We are just beginning to understand the extensive epigenetic and functional resemblances between early developmental stages and cancer where transposable elements play a pivotal role (Table 2).

**TABLE 2:** Genes and interacting miRNAs involved in the shared features of PGCs and CSCs.

	Common features of PGCs and CSCs	Involved genes	Interacting miRNAs	References PGCs	References CSCs
1	Gametic recapitulation	TRIM71 MEIOB SPATA22 LIN28A and B	miR-7 miR-202 Let-7	Bruggeman et al. (2018), Irie et al. (2015)	Bruggeman et al. (2018), Cubela, 2008; Chen et al. (2022), Bruggeman et al. (2023)
2	Active retrotransposons	LINE 1	Let-7a	Jachowicz et al. (2017)	Ohms et al. (2014)
3	Immortalization	SOX2, POU5F1 NANOG	miR-17∼92 cluster	Heaney et al. (2012), Wu et al. (2014)	Mendell (2008)
4	ZGA-like cell reprogramming	DUX4 SOX2, POU5F1 NANOG	miR-675 miR-17∼92 cluster miR-371–373	Karlsson et al. (2021) Rosa and Brivanlou (2017), Wu et al. (2014)	Smith et al. (2023) Cioffi et al. (2015), Ventura et al. (2008)
5	Aerobic glycolysis (Warburg –like metabolism)	HIF1A HK2	miR-18a-5p miR-29b miR-210-3p	Oliveira et al. (2014)	Wu et al. (2021)
6	Unchecked cell proliferation	CCND1	miR-302/367 cluster miR-371–373	Laurent (2008)	Montalto and De Amicis (2020)
7	Meiosis	MEIOB SPATA22 LIN28A TRIM71	miR-202 miR-7 Let-7	Wang et al. (2016a)	Chen et al., 2022; Cubela (2008)
8	Induction of neoangiogenesis	CCND1	miR-371–373	Yasui et al. (2006)	Wang et al. (2018)
9	Immune evasion	HLA-G	mir-148a miR-152	Yao et al. (2014), Manaster et al. (2012)	Lin and Yan (2015)
10	Migratory capabilities	NANOS3	miR-430	Wang et al. (2022), Angeles Julaton and Reijo Pera (2011), Chuai et al. (2012)	Zhang et al. (2020a), Grelet et al. (2015)
11	Fusion capabilities (fusogens)	IZUMO1 ERVW-1	none	Shimono et al. (2009), Morales-Prieto et al. (2012)	Choi et al. (2011)

Transposable elements, also known as jumping genes, can move from one genomic locus to a new one and represent almost half of the human genome. Retrotransposons account for about 42% of the human genome and use an RNA intermediate to move to new genomic locations. They contribute to the genetic polymorphisms being regarded as important drivers of genome evolution through acquisition of new functional characteristics from homing to new coding regions of the genome. Transposable elements are almost always completely inactivated in healthy somatic cells (Walsh et al., 1998). While epigenetic inactivation/silencing of transposable elements through histone methylation and deacetylation occurs in differentiated cells to circumvent their potential genotoxic effect, the primordial germ cells have active retrotransposons responsible for their specification and ensuring genetic variability in the future zygote (Xiang et al., 2022). Human Endogenous Retroviruses (HERV) retrotransposons, vestiges of an ancestral retrovirus infection in the germ line, are still being actively transcribed in the placenta, hypothalamus, and testis (Crowell and Kiessling, 2007; Kim et al., 2004).

PGCs are precursors of both male and female germ cells. One representative epigenetic reprogramming event in PGCs is the global demethylation during their specification, which also puts transposable elements into a transcriptionally active state (Lesch and Page, 2012). In a subsequent step, another DNA demethylation event takes place in a locus-specific manner to induce meiotic genes, germline genes required for gamete generation and imprint erasure (Yamaguchi et al., 2012; Hackett et al., 2012).

In embryogenesis, after sperm-egg fusion, the resulting zygote undergoes global demethylation (Altun et al., 2010). During this reprogramming, some transposable elements keep their methylated state, similar to the maintenance of methylation of imprinted genes while others demethylate initially but soon remethylate through recruitment of the *de novo* methylation apparatus (Theunissen et al., 2016). However, some transposable elements remain unmethylated and become transcriptionally active at this early stage regulating essential totipotency and pluripotency factors (Rodriguez-Terrones and Torres-Padilla, 2018). Furthermore, active transposable elements of the placenta regulate key transcripts that are essential for invasion, immune modulation, neoangiogenesis, growth and proliferation, the placenta showing a DNA methylation landscape resembling that of tumors (Robinson and Price, 2015).

The repetitive nature of retrotransposons can result in incorrect recombination events and lead to translocations, deletions and insertions, resulting in genomic instability, a hallmark of cancer (Hollister and Gaut, 2009). Long interspersed element 1 (LINE-1) is a highly active retrotransposon in the primordial germ cells being involved in key epigenetic reprogramming events of meiosis and gametogenesis (Zhou et al., 2022). LINE-1 overexpression and retrotransposition are hallmarks of cancers, promoting genetic heterogeneity in tumors (Mendez-Dorantes and Burns, 2023).

Because their mobilization can result in deleterious effects, as highlighted by retrotransposon (RE)-associated diseases (Belancio et al., 2009; Payer and Burns, 2019), cells have developed diverse mechanisms to repress their activity, primarily by epigenetic silencing, which includes DNA (Haggerty et al., 2021) and histone (Lindehell et al., 2023) methylation. *De novo* somatic insertions of the L1 REs have been observed in particular in colon epithelial cells (Nam et al., 2023) and cortical neurons from both diseased and normal brain tissues, with potential implications in the genomic diversity of neurons for the later (Zhao et al., 2019). Furthermore, retrotransposition events have been shown to contribute to hippocampal genomic variations and cognition processes (Bachiller et al., 2017). Several lines of evidence indicate that neuronal progenitor cells seem to be a “hot spot” of retrotransposition activity in the brain (Coufal et al., 2009; Muotri et al., 2005), linking the pluripotent phenotype of cells to an increase in retrotransposition events. Whole genome methylation analysis suggests that a decrease in methylation of L1 promoter occurs early in embryogenesis, reaching the lowest degree in gastrulation stage of the embryo. Post-gastrulation remethylation and subsequent L1 silencing is associated with the differentiation and maturation of the embryo and continues throughout life. In elderly an increase in retrotransposition events varies depending on the genetic imprinting inherited from parents, potentially leading to malignant transformation of cells (Nam et al., 2023). Because genomic instability is a hallmark of cancer (Negrini et al., 2010), and REs represent a major source of genomic instability (Daskalos et al., 2009), it can be reasonable to say REs are important drivers of cancer pathogenesis, and high levels of retrotransposition is associated with cancer cell stemness (Apostolou et al., 2015) a feature of highly aggressive tumors, relapse and resistance to treatment (Chengizkhan et al., 2020).

The undifferentiated stem cell phenotype, present in multiple tissues, and early in development, might be a consequence of REs activity. Therefore, genes that are regulated by REs in stem cells might overlap with the ones that are regulated by REs in tumor cells. It is known that regulation of the transcriptome in embryogenesis resembles the one found in malignant tumors, as reveled by a comprehensive analysis of expression data from 1,094 individual arrays derived from a broad spectrum of tumors. Clustering in relation to a developmental timeline expression pattern, resulted in three groups. Group 1 includes tumors enriched in genes upregulated in early embryogenesis, and related to stem cells phenotype, and downregulated genes linked to tissue specificity and development. Group 3 comprise tumors with gene expression patterns mostly related to the late development timeline, while group 2 falls in between. It is worth mentioning that group 1 comprised tumors with an aggressive phenotype and high proliferating rates, such as lung cancer. Nonetheless, a core gene set that map to early development was identified in approximately 50% of the tumor expression data analyzed (Naxerova et al., 2008).

As a link between the early embryogenesis stages and malignancy is highly probable to exist, it remains to question if REs are the ones that might drive these similar phenotypes of embryogenesis and tumorigenesis, yet different as we perceive them to be. A recent study points to such an assumption, showing that knockout of the L1 retrotransposons in mouse embryos lead to developmental arrest, while induced L1 expression is associated with upregulation of distant genes essential for zygotic activation and transition to blastocyst, in a transcriptional-dependent manner (Naxerova et al., 2008), rather than chromatin remodeling as a previous study suggest (Jachowicz et al., 2017). Therefore, if retrotransposon transcripts, such L1, might induce zygotic activation after fecundation, the question that might arise is related to the origin of these transcripts. The answer could possibly reside in the transcriptomic profile of the spermatozoa and oocytes. RNA sequencing analysis non-coding small non-coding RNAs (sncRNAs) from human mature sperm reveals that approximately 65% of transcript reads belong to repeat sequences. Among the known repeats, the LINE, LTR and SINE retrotransposons are the most prevailing ones. The remainder of the sncRNAs are represented by regulatory RNAs, such piwi interacting RNAs (piRNAs) and microRNAs (miRNAs) (Krawetz et al., 2011). In addition, an abundance of coding mRNAs has also been identified, with functions in transcription regulation and cell cycles, among other (Dadoune et al., 2005). The transcriptome of the human oocytes also shows expression of retrotransposons, though this study points to an abundance of LTR and SVA retrotransposons as compared to LINE (Georgiou et al., 2009). It would be expected that differentiated cells, such as sperm cells and oocytes, would exhibit low levels of retrotransposons expression. Papers point to a genome-wide demethylation in the initial stages of sperm development, while the mature sperm cells exhibit an elevated methylation level and hence reduced transcriptional activity (Deniz et al., 2019). This implies an accumulation of retrotransposition transcripts in the absence of transcriptional activity in mature germ cells, probably to limit the mutagenic potential of REs. After fecundation, the transcriptomic profiles of the germ cells bring the regulatory RNAs in one cell, the zygote, to initiate the embryonic development. It might be that the differences in the retrotransposon transcriptomic profile confer the germ cells different proprieties. It is known that syncytins derived from the envelope of HERV retrotransposons confer fusogenic properties to the cells encoding them and are involved in placentation of the mammalian embryos. A higher level of LTR retrotransposons-derived syncytins expression in the oocytes (Georgiou et al., 2009) could make them susceptible to selective fusion with the sperm cells, as oocytes are not produced in their millions compared to sperm cells and are hence less likely to produce a wide-spread syncytium and infertility. The more abundant LINE retrotransposons in the sperm cells, alongside with the other regulatory transcripts (Krawetz et al., 2011), would mediate activation of pluripotent phenotype in the new zygote cell, and proliferation to multiple cells that later would suffer the process of differentiation in specific tissues following an epigenetic pattern of regulation.

Retrotransposons were initially regarded as “junk” DNA and later as drivers of evolution, but now they can be safely considered as important regulators of development, providing the cell with pluripotent characteristics. Such a phenotype is mandatory, both in early stages of embryogenesis, and later in adulthood.

As transposable elements activate early developmental genes, they can also be key contributors to the malignant transformation of the cell. A mechanism similar to the cell fusion in fecundation where two terminally differentiated cells bring together different transcriptomic profiles, that initiate the expression of a pluripotent phenotype, and lead to cell proliferation and growth, might also be responsible for triggering early cancer pathogenesis. Furthermore, the same mechanisms that protect the developing embryo from the mother’s immune system might also help cancer cells to evade immune surveillance, as studies would suggest (Zhu et al., 2021).

Transcriptionally active retrotransposons are seen in CSCs and drive upregulation of numerous oncogenes by their endogenous promoter activity in a process termed onco-exaptation (Lynch-Sutherland et al., 2020; Jang et al., 2019). While the mechanisms of retrotransposons activation in cancer is unknown, the epigenetic landscape in cancer resembles that of early development with transposable elements activation, hence dedifferentiation has been proposed as a potential mechanism of retrotransposon demethylation and reawakening in tumor cells (Lynch-Sutherland et al., 2020). However, more recent investigations using single-cell transcriptomics of stem vs. differentiated and cancer vs. normal cells indicates a rather atavistic type of genomic signature in cancer stem cells rather than the result of dedifferentiation, putting the zygote-like cancer stem cell (unicellular-like state) as a central hallmark of cancer (Vinogradov and Anatskaya, 2025). How this unicellular-like state is acquired in cancer stem cells is still unknown. Since sperm and eggs fuse together selectively and not randomly with any other cell type, the odd possibility of unselective fusion triggering subsequent reactions similar but ectopic to those occurring in normal fecundation could bring together all the hallmarks of cancer into one unified theory. This is supported by recent findings that spontaneous differentiation of germ cells from human embryonic stem cells has been shown to occur *in vitro* (Geijsen and Daley, 2008) and while doubtful, we cannot exclude such an occurrence *in-vivo* with pPSCs (e.g., VSELs) as protagonists. In this context, unselective stem cell fusion leading to the emergence of a zygote-like cancer stem cell becomes even more intriguing since fertilizing oocytes using artificial fusion with somatic cells as male germ cells has already been demonstrated (Lacham-Kaplan et al., 2001).

Little is known about the potential acquisition of demethylated/active retrotransposons from fusion events. As germ cells carry active retrotransposons required for propagating themselves and ensuring genetic diversity in the future zygote (Zhou et al., 2022), we suspect that a putative fusion of a PGC-related pPSC in postnatal tissues with a mutation-harboring somatic cell for cell rescue could potentially explain the acquisition of active retrotransposons in the resulting ZL-CSC. This could be responsible for tumor heterogeneity as seen in many cancers. Nonetheless, global demethylation following their fusion, as seen in the selective fusion that leads to zygote formation, could also explain the imprint erasure in a zygote-like cell resulting from unselective fusion. Regardless of their mechanism of activation, retrotransposons remain a distinctive feature in both embryogenesis and oncogenesis and their demethylation/activation mechanisms in cancer warrants further investigation.

## 6 The proposed germ-somatic fusion hypothesis and testable implications

The establishment of alternative concepts to explain the cell of origin of cancer is warranted, as survival rates in last stage cancer have seen only marginal improvement over the past century (Liu, 2022). The emergence of a new tide of genomics data from cancer genome analysis in recent years points toward copy number and genomic structural changes that are inconsistent with the conventional somatic mutation theory (Campbell et al., 2020). Next-generation sequencing data indicates that in tumorigenesis, the genomic rearrangements are highly improbable to have accumulated over time and instead holds that nearly all mutations occur during a one-off cellular crisis, promoting the evolution toward cancer (Stephens et al., 2011). Following the avalanche of big genomic data, many parallels between development and the gametogenic program induction in cancer have been drawn in the last decades. The identification of cancer-testis - and trophoblast specific - antigens in tumors have generated provocative concepts, viewing cancer as a “somatic pregnancy” (Old, 2007) or “chaotic embryogenesis”- based on McClintock’s heredity (Liu, 2022). In an elegant tumor’s gametogenesis-related model, Liu et al. provides consistent arguments based on experimental data suggesting that the formation of tumor cells is in some way similar to fertilization, with PGC-like cells likely being an origin for somatic cancer initiation and metastasis via stage arrest resulting in tumors with immature tissues, and parthenogenetic activation of primary oocyte at meiotic arrest causing tumors with mature tissues (Liu C. et al., 2022). Another mechanism of tumor formation holds that somatic cells can react to stress by increasing cell size, restructuring the genome for reproduction or neoplastic transformation and explaining the occurrence of polyploid giant cancer cells (PGCCs) in advanced tumors. This genomic restructuring not only creates a complex genotype but also activates an embryonic program forming the blastomere- or blastocyst-like embryo, since they resemble the pre-implantation embryo in morphology, gene expression, and the ability to generate germ cell tumors (Liu, 2022).

While these intriguing theories rely on the spontaneous activation of alternative developmental programs to generate pluripotent-like cellular states without zygotic fusion events, our concept distinguishes this model from alternative no-fusion mechanisms holding that activation of the developmental programme implies two cells involved in a failed fusion-driven cell rescue when the responders to cell stress signals are the primitive germline related stem cells. A consequence of such failed attempt of cell rescue by unselective gametic-like fusion could lead to recapitulation of the zygotic program in tumors and metastasis which seems to follow specific homing patterns to predefined niche - as in primitive cell migration during development and fetal-maternal microchimerism-rather than just random spread, in a tumor-host microchimerism-like manner.

**FIGURE 3:**
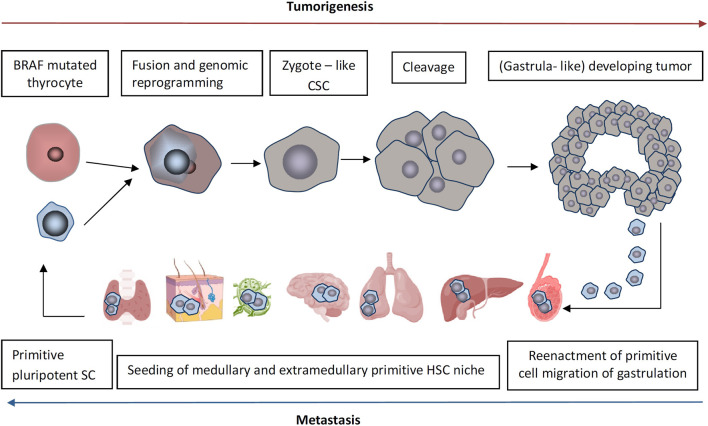
The proposed cycle of tumorigenesis and metastasis centering the zygote-like cancer stem cell (ZL-CSC) and exemplified for the case of an anaplastic thyroid carcinoma (ATC) as a representative model for solid tumor transformation. We speculate that primitive (germline related) pluripotent stem cells (pPSCs) originating from migratory events of gastrulation (Carnegie stage 8) and residing in the steady state in primitive hematopoietic stem cell (HSC) niche of postnatal tissues, can be recruited via CXCL12/CXCR4 chemokine axis for the repair of mutation-harboring somatic cells signaling distress via SASP cytokines. Fusion of quiescent pPSCs with the damaged cell can lead to somatic cell nuclear reprogramming to a totipotent-like state similar to the zygote but with a high rate of developmental abnormalities caused by epigenetic remnants that alter gene expression regulation in the resulting ZL-CSC. Still, the ectopic gastrula-like/tumoroid-like developing tumor (subclinical stage) recapitulates faultily the early Carnegie stages of embryo development, with primitive cell migration and early metastatic spread resembling migratory events of gastrulation and fetal-maternal microchimerism. By recapitulation of fetal-maternal microchimerism, the cancer cell migration suggests that cancer cells don’t spread randomly but instead are homing to specific environments that support primitive stem cells. As the most lethal ATC often transforms from differentiated thyroid cancer the poorly understood but complex intratumor transformation process could result from a repeated cycle of tumorigenesis involving pPSCs fusion events.

The migratory events of distant metastatic spread are reflected in the phenomenon of fetal-maternal microchimerism where primitive stem cells of the developing embryo are able to seed not only fetal structures but also various maternal tissues during gastrulation, persisting in the bone marrow and other primitive stem cell niche, in the steady state as tissue-resident pluripotent stem cells throughout the mother’s life with implications in both health and disease (Cismaru et al., 2018; Cismaru et al., 2019). While pluripotent stem cells have been identified in various post-natal tissues and have been labeled as endothelial progenitor cells (EPCs), multipotent adult progenitor cells (MAPCs), marrow-isolated adult multilineage inducible cells (MIAMIs), multipotent adult stem cell (MACS), fetal microchimeric stem cells (FMSCs) or very small embryonic-like stem cells (VSELs) (Cismaru et al., 2019; Ratajczak et al., 2019; Ratajczak et al., 2008) depending on the researcher groups, it is likely that in most cases, similar or overlapping populations of post-migratory and quiescent tissue resident pPSCs have been described since their molecular signature supports their epiblast/germ line origin (Shin et al., 2010). Recapitulation of migratory events of gastrulation in tumorigenesis and their striking resemblance with fetal-maternal microchimerism lead us to hypothesize that following a putative fusion of a quiescent tissue resident pPSC with a mutation-harboring somatic cell for cell rescue, the nuclear transfer may potentially lead to genomic reprogramming and ZL-CSC emergence followed by passage through early Carnegie stages with the reenactment of the migratory events of early development both in the ectopically developing embryoid body-like tumor as well as in host tissues, mimicking the fetal-maternal microchimerism previously explored by our group (Cismaru et al., 2019) (Figure 3).

As a representative model for solid tumor transformation, anaplastic thyroid cancer (ATC), while being the deadliest type of undifferentiated thyroid malignancy, reflects many of the aspects related to the activation of the zygotic genome (DeSouza et al., 2025) and retroelement reactivation in disease (D’Alessandre et al., 2025). Since synchronous differentiated thyroid cancer regions have been reported on histopathology in about 70% of patients with ATC this suggests that it is dedifferentiation of DTC that leads to progression to ATC (Santarpia et al., 2008; Molinaro et al., 2017). However, the passage from a diploid stage to an aneuploid genotype and transition to a mesenchymal phenotype over the course of progression from DTC to ATC as revealed by single-cell transcriptomics (Lu et al., 2023) suggests that another mechanism for unlocking the zygotic genome in ATC could be fusion-driven (Figure 3).

Erasure of the gametic imprinting in PGC/germline-related pPSCs could bring more insights into how tumorigenesis may revoke some of the early stages of embryonic development. It becomes intriguing to assume that the ontogeny of stem cells originating in more primitive tissue-resident precursors such as PGC-related pPSCs could have crucial implications for the fate of the resulting hybrid cell that may be amenable to revoking specific stages of embryogenesis ectopically (Cismaru et al., 2022; Ratajczak et al., 2019). This is since PGCs were shown to be able to undergo chromatin reorganization and imprint erasure with sequential epigenetic changes and genome-wide DNA demethylation to reset the epigenome for totipotency (Hackett et al., 2012). Following a putative fusion, nuclear transfer and genomic reprogramming from a PGC-related pPSC to a mutation-harboring cell, this would potentially result in activation of early developmental genes and zygote-like cell formation but faultily, similar to that reported in the cloning technique with non-enucleated oocytes (Rouillon et al., 2019). This implies that oncogenesis would presumably imply a deleterious and ectopic embryogenesis-like process without the classical fecundation trigger. As fecundation has been the *de facto* trigger of embryogenesis in the classical dogma of fertilization, research in the last decades has shown that embryogenesis can also occur in the absence of sperm-egg fusion using the nuclear transfer technique (Lacham-Kaplan et al., 2001). However this requires an external manipulation of the cells such as the one used in cloning by nuclear transfer from a somatic cell in an enucleated oocyte leading to reactivation of genes previously inactivated by cell differentiation and triggering embryogenesis (Tian et al., 2003). Furthermore, non-enucleated oocytes have also been successfully used in the nuclear transfer technique leading to the disappearance of the oocyte DNA (Rouillon et al., 2019).

**FIGURE 4:**
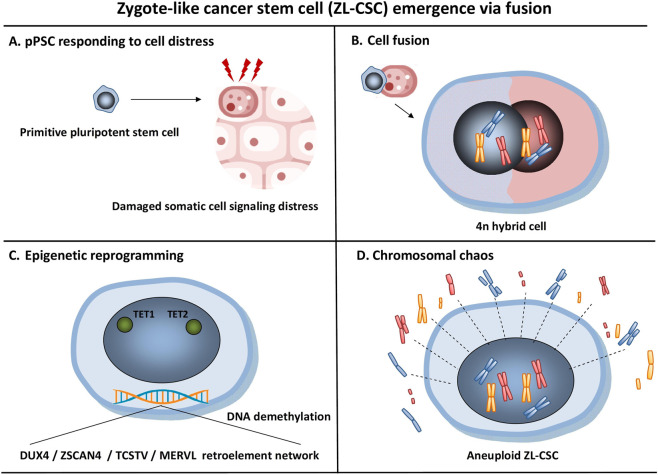
Step-by-step diagram illustrating the core cellular and molecular mechanisms of a failed cell rescue mechanism by fusion, leading to epigenetic reprogramming and emergence of a ZL-CSC with chromosomal chaos. **(A)** Primitive pluripotent stem cell responding to damaged somatic cell distress; **(B)** Fusion and formation of a 4n hybrid cell; **(C)** Epigenetic reprogramming with DNA demethylation and activation of zygotic gene networks; **(D)** Chromosomal chaos producing an aneuploid cancer stem cell.

Numerous concepts have been developed over the last years that tried to explain the origin of the cancer cell and the genetic and epigenetic mechanisms regulating the gene expression related to increased proliferation, blocked apoptosis, evasion of immune reactivity and preparation of metastasis and spread. Several works exploring the origin of CSCs suggest that they may arise by VSELs malignant transformation (Bhartiya et al., 2024; Sharma and Bhartiya, 2021). While such pPSCs in postnatal tissues seem to be involved in tumorigenesis, we speculate that the mechanism of their malignant transformation is not spontaneous as consequence of environmental insults but rather fusion driven since further tumor development closely resembles early embryogenesis whereas metastasis resembles fetal-maternal microchimerism. Our concept implies that although the cancer originates from a single cell - the ZL-CSC, tumorigenesis involves two types of cells, one pluripotent with germ line traits–the tissue resident quiescent PGC/germline-related pPSC, and one somatic but damaged by environmental insults, which fuse together for somatic cell rescue but produce the ZL-CSC through nuclear reprogramming akin to an unselective fecundation (Figure 4).

**FIGURE 5:**
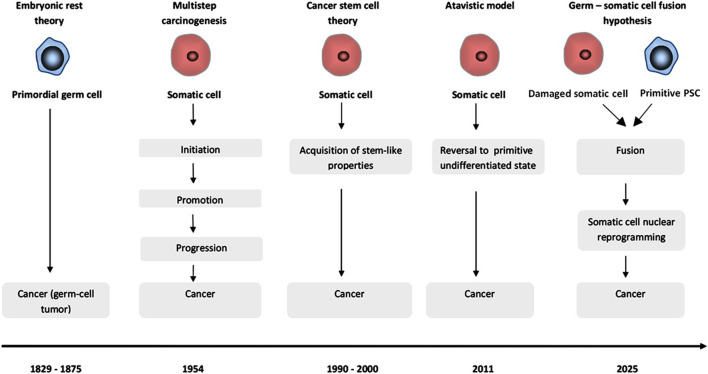
Graphical representation of the evolution of oncogenesis models over time including the currently proposed concept of germ-somatic cell fusion hypothesis.

It becomes intriguing to speculate that a spontaneous nuclear transfer by unselective cell-cell fusion could trigger a fecundation-like mechanisms leading to activation of embryonic developmental pathways such as Wnt, Notch, Hedgehog (Collu et al., 2014) (Figure 2), but ectopic and defectuous, with recapitulation of preimplantation and implantation stages, and even cell migration and tumor-host microchimerism at least up to a point, yet such a hypothesis has not been previously proposed explicitly (Figure 5).

Regarding the testable implications of our concept, potential discriminative prediction assays can clarify the emergence mechanisms of ZL-CSCs and differentiate germ-somatic fusion mechanisms from non-fusion reprogramming such as dedifferentiation or parthenogenesis. Assessing the genomic imprinting status using single-cell methylome sequencing, allele-specific RNA-seq and phasing single nucleotide polymorphisms (SNPs) can discriminate between parental genomes. Biparental imprinting marks and no genome-wide uniparental disomy pattern can indicate a fusion driven ZGA transcriptional reprogramming rather than parthenogenesis mechanisms which is associated with global skew toward maternal methylation patterns with regions of uniparental isodisomy. Moreover, single-cell whole-genome sequencing and mitochondrial haplotype tracing studies that show mixed SNP haplotypes from two clones and discordant mitochondrial genotypes can predict ZL-CSCs emerge via fusion. Otherwise, dedifferentiation would show a genotype that matches parental clone and no new haplotype combinations. CRISPR-Cas9 based barcode lineage tracing can further differentiate fusion from other non-fusion mechanisms of ZL-CSCs emergence (Kester and van Oudenaarden, 2018) to strongly resolve the proposed model.

## 7 Conclusion

The concept of CSCs lays at the basis of tumor heterogeneity but the exact mechanism of their occurrence is still incompletely elucidated. Stem cell fusion has been described more than 130 years ago but has been in the focus of research only recently since it represents a common occurrence in cancer and also in mutation-harboring nonmalignant tissues. As accumulating data suggests, this cell rescue mechanism could repair highly functional cells and even reprogram them through a cloning-like process endowing the resulting hybrids with new capabilities that result from nuclear transfer. While stem cell fusion can bring new insights into the mechanisms that lead to the emergence of cancer stem cells, there are still missing pieces in the puzzle of tumor development since this repair mechanism by fusion has been shown to promote cell differentiation and restoration of normal replication and apoptosis in cancer cells (Blau, 2002). What could trigger the generation of totipotent ZL-CSCs that recapitulate embryogenesis is not completely understood but may depend on the suspected ontogeny of the fusing cells.

The basic hypothesis of this speculative analysis and emerging notion is that stem cell fusion attempts the restoration of genetic abnormalities in functional cells which brings a new set of integral genes but can also induce a cloning-like genomic reprogramming and abnormal histogenesis, if germline-related pluripotent stem cells become involved in the rescue process. When quiescent tissue resident germ line related pPSCs attempt to rescue mutation-harboring cells by fusion, a fecundation-like cell reprogramming could potentially occur with the emergence of zygote-like/unicellular-like CSCs that demethylate their developmental meiotic genes. This speculative concept of oncogenesis becomes appealing for both solid and hematological malignancies since it corroborates past and current hallmarks of cancer into one unified theory, opening new avenues for cancer research and therapy. To our knowledge, this concept has not been proposed explicitly.

Implantation of a ZL-CSC in the host tissue should induce invasion, immune tolerance, neoangiogenesis and distant spread which is not much different from the reactions triggered by a developing embryo outside the uterus in ectopic tissues such as the fallopian tubes and more rarely in the ovary, cervix, or outside the uterus and its annexes in the abdominal cavity in omentum, intestines, liver, spleen, lesser sac, and retroperitoneal space (Agarwal et al., 2014; Ren et al., 2022).

Specific fusogens such as syncytins and IZUMO1 involved in physiological fusions, are likely involved in the pPSC-somatic fusion in postnatal tissues since these fusogens are also expressed in multiple tumor types (Wang et al., 2023). In normal trophoblast formation and placentation, cell fusion leads to genomic alterations and extensive mutation (Coorens et al., 2021). However, while multiple mechanisms can stabilize the genome and ensure healthy and coordinated development in the post-implantation embryo, a failed fusion driven cell rescue could have chromoplexy and chromothripsis as drivers of unstable genomic changes in cancer development (Stephens et al., 2011; Liu, 2022).

How does the fusion evade immune detection, and how does it differ from physiological fusion events like trophoblast formation is still an area of active research. Following genomic reprogramming and activation of the zygotic genome in the resultant hybrid, the ZL-CSC appears to hijack the developmental programme for its own growth and immunologic acceptance. With the experimental evidence of fusion events in human tumors (Wang R. et al., 2016; Rengstl et al., 2013; Dittmar et al., 2009), it is becoming increasingly difficult to escape the conclusion that the cellular crisis leading to activation of the embryonic programme might not be spontaneous in a single cell, but could potentially involve a two cell fusion-driven reprogramming, akin to development.

Whether somatic mutations are the root cause of cancer or merely its consequence is still debatable. Quiescent stem cells are not expected to harbor somatic mutations but cancer cells with genomic instability can acquire somatic mutation following their clonal expansion. When coding and noncoding genomic elements are taken into account, cancer genomes typically feature four to five driver mutations; nevertheless, 5% of malignant tumors have none at all (Campbell et al., 2020). Aging is correlated with accumulation of somatic mutant clones and the activation of oncogenic mutations (Martincorena et al., 2018), indicating that a certain amount of genomic instability can be controlled during both development and aging. Since driver mutations are often seen in benign tissue, they seem necessary but not sufficient to trigger oncogenesis. Perhaps fusion is the second hit leading to tumor transformation. Our speculation is that fusion of a germ line related quiescent stem cell with a mutation-harboring somatic cell is what might actually induce reprogramming and genomic instability in the resulting CSC. Therefore, our hypothesis is that mutation in the somatic cell is not only the trigger but also a consequence of altered gene expression regulation perpetuated by fusion in tumorigenesis. This offers a necessary paradigm shift in cancer treatment, from destroying a mutant cell to stopping a rogue developmental program. The overlap between embryogenesis and oncogenesis is undeniable so understanding the very start of life is essential to cure the disease that so often ends it.

Currently there is no direct observation tracking how ZL-CSCs emerge. However, due to the role of transposable elements in development and their interactions with meiotic genes and pluripotency factors such as OCT4 and NANOG, SOX2, PROM1, along with their transcriptionally active state in PGCs, and the activation of such factors in many cancers, we believe our hypothesis is worth pursuing, warranting further investigation. Experimental validation of this concept could challenge current cancer origin models, link developmental biology and oncology in a novel way and create premises for anti-reprogramming therapies.

It remains a challenge to define the role of cell rescue by stem cell fusion, whether it is primarily reparative or oncogenic. Because such type of cell rescue correlates with both favorable and unfavorable biological outcomes, this suggests that the process might be a recent evolutionary event that could still be ongoing. As it takes millions of years for evolution to complete, we might have the rare opportunity to behold a new trend in maintaining genetic integrity in somatic cells by “intracellular chimerism”.

## Data Availability

The original contributions presented in the study are included in the article/supplementary material, further inquiries can be directed to the corresponding author.

## Glossary

CSCs: cancer stem cells
ZL-CSCs: zygote-like cancer stem cells
PSCs: pluripotent stem cells
Primitive: originating during early embryogenesis
pPSCs: primitive pluripotent stem cells
VSELs: very small embryonic-like stem cells
Gametic recapitulation: re-establishment of gamete-specific DNA methylation.
Fetal-maternal microchimerism: long-term homing of fetal stem cells in maternal niche
TE: transposable elements
Onco-exaptation: new regulatory functions of transposable elements in cancer cells

## References

[B1] Abdelbaset-Ismail A. Suszynska M. Borkowska S. J. Adamiak M. Ratajczak J. Ratajczak M. Z. (2015). Human hematopoietic stem/progenitor cells (HSPCs) and mesenchymal stromal cells (MSCs) express several functional pituitary and gonadal sex hormone receptors - identification of follicle stimulating hormone (FSH) and luteinizing hormone (LH) as new growth factors for HSPCs and MSCs. Blood 126, 2393. 10.1182/BLOOD.V126.23.2393.2393

[B2] Agarwal N. Odejinmi F. Frcog M. (2014). Early abdominal ectopic pregnancy: challenges, update and review of current management. Obstet. Gynaecol. 16, 193–198. 10.1111/TOG.12109

[B3] Altun G. Loring J. F. Laurent L. C. (2010). DNA methylation in embryonic stem cells. J. Cell. Biochem. 109, 1–6. 10.1002/JCB.22374 19899110 PMC3289679

[B4] Aman A. Piotrowski T. (2010). Cell migration during morphogenesis. Dev. Biol. 341, 20–33. 10.1016/J.YDBIO.2009.11.014 19914236

[B5] Angeles Julaton V. T. Reijo Pera R. A. (2011). NANOS3 function in human germ cell development. Hum. Mol. Genet. 20, 2238–2250. 10.1093/HMG/DDR114 21421998 PMC3090199

[B6] Antony J. M. Van Marle G. Opii W. Butterfield D. A. Mallet F. Yong V. W. (2004). Human endogenous retrovirus glycoprotein–mediated induction of redox reactants causes oligodendrocyte death and demyelination. Nat. Neurosci. 710 (7), 1088–1095. 10.1038/nn1319 15452578

[B7] Apostolou P. Toloudi M. Chatziioannou M. Kourtidou E. Mimikakou G. Vlachou I. (2015). Involvement of retrotransposon L1 in stemness and cellular plasticity. Cell Commun. Adhes. 22, 1–7. 10.3109/15419061.2014.970270 25327441

[B8] Bachiller S. del-Pozo-Martín Y. Carrión Á. M. (2017). L1 retrotransposition alters the hippocampal genomic landscape enabling memory formation. Brain. Behav. Immun. 64, 65–70. 10.1016/j.bbi.2016.12.018 28012829

[B9] Bae J.-S. Han H. S. Youn D.-H. Carter J. E. Modo M. Schuchman E. H. (2007). Bone marrow-derived mesenchymal stem cells promote neuronal networks with functional synaptic transmission after transplantation into mice with neurodegeneration. Stem Cells 25, 1307–1316. 10.1634/stemcells.2006-0561 17470534

[B10] Bartnicki E. Yeung S. Damani-Yokota P. Khanna K. (2024). Nerve- and airway-associated interstitial macrophages regulate anti-tumor CD8 T cell immunity in tumors in the lung. J. Immunol. 212, 1235–6121. 10.4049/JIMMUNOL.212.SUPP.1235.6121

[B11] Batlle E. Clevers H. (2017). Cancer stem cells revisited. Nat. Med. 23, 1124–1134. 10.1038/NM.4409 28985214

[B12] Beck B. Blanpain C. (2013). Unravelling cancer stem cell potential. Nat. Rev. Cancer 13, 727–738. 10.1038/NRC3597 24060864

[B13] Belancio V. P. Deininger P. L. Roy-Engel A. M. (2009). LINE dancing in the human genome: transposable elements and disease. Genome Med. 1, 97. 10.1186/GM97 19863772 PMC2784310

[B14] Berthet C. Kaldis P. (2007). Cell-specific responses to loss of cyclin-dependent kinases. Oncogene 26, 4469–4477. 10.1038/SJ.ONC.1210243 17297466

[B15] Bhartiya D. (2025). Will detecting somatic mutations three years prior to diagnosis help early prediction of cancer? Stem Cell Rev. Rep. 21, 2807–2809. 10.1007/S12015-025-10954-0 40789791

[B16] Bhartiya D. Raouf S. Pansare K. Tripathi A. Tripathi A. (2024). Initiation of cancer: the journey from mutations in somatic cells to epigenetic changes in tissue-resident VSELs. Stem Cell Rev. Rep. 20, 857–880. 10.1007/S12015-024-10694-7 38457060

[B17] Bianchi E. Doe B. Goulding D. Wright G. J. (2014). Juno is the egg izumo receptor and is essential for mammalian fertilization. Nature 508, 483–487. 10.1038/NATURE13203 24739963 PMC3998876

[B18] Bielski C. M. Zehir A. Penson A. V. Donoghue M. T. A. Chatila W. Armenia J. (2018). Genome doubling shapes the evolution and prognosis of advanced cancers. Nat. Genet. 50, 1189–1195. 10.1038/S41588-018-0165-1 30013179 PMC6072608

[B19] Bittner R. E. Schöfer C. Weipoltshammer K. Ivanova S. Streubel B. Hauser E. (1999). Recruitment of bone-marrow-derived cells by skeletal and cardiac muscle in adult dystrophic mdx mice. Anat. Embryol. Berl. 199, 391–396. 10.1007/s004290050237 10221450

[B20] Bjerregaard B. Holck S. Christensen I. J. Larsson L. I. (2006). Syncytin is involved in breast cancer-endothelial cell fusions. Cell. Mol. Life Sci. 63, 1906–1911. 10.1007/S00018-006-6201-9/METRICS 16871371 PMC11136146

[B21] Blau H. M. (2002). Stem-cell fusion: a twist of fate. Nat 2002, 437. 10.1038/419437a 12374136

[B22] Bruggeman J. W. Koster J. Lodder P. Repping S. Hamer G. (2018). Massive expression of germ cell-specific genes is a hallmark of cancer and a potential target for novel treatment development. Oncogene 37, 5694–5700. 10.1038/S41388-018-0357-2 29907769 PMC6193945

[B23] Bruggeman J. W. Koster J. van Pelt A. M. M. Speijer D. Hamer G. (2023). How germline genes promote malignancy in cancer cells. BioEssays 45, 2200112. 10.1002/BIES.202200112 36300921

[B24] Brukman N. G. Uygur B. Podbilewicz B. Chernomordik L. V. (2019). How cells fuse. J. Cell Biol. 218, 1436–1451. 10.1083/JCB.201901017 30936162 PMC6504885

[B25] Campbell P. J. Getz G. Korbel J. O. Stuart J. M. Jennings J. L. Stein L. D. (2020). Pan-cancer analysis of whole genomes. Nature 578, 82–93. 10.1038/s41586-020-1969-6 32025007 PMC7025898

[B26] Carvalho A. A. Martelli D. R. B. Carvalho M. F. A. Swerts M. S. O. Júnior H. M. (2021). Cafe-au-lait spots as a clinical sign of syndromes. Res. Soc. Dev. 10, e14310917607. 10.33448/RSD-V10I9.17607

[B27] Casanova-Acebes M. Dalla E. Leader A. M. LeBerichel J. Nikolic J. Morales B. M. (2021). Tissue-resident macrophages provide a pro-tumorigenic niche to early NSCLC cells. Nat 2021, 578–584. 10.1038/s41586-021-03651-8 34135508 PMC8923521

[B28] Chen J. F. Mandel E. M. Thomson J. M. Wu Q. Callis T. E. Hammond S. M. (2006). The role of microRNA-1 and microRNA-133 in skeletal muscle proliferation and differentiation. Nat. Genet. 38, 228–233. 10.1038/ng1725 16380711 PMC2538576

[B29] Chen K. Huang Y. H. Chen J. L. (2013). Understanding and targeting cancer stem cells: therapeutic implications and challenges. Acta Pharmacol. Sin. 34, 732–740. 10.1038/aps.2013.27 23685952 PMC3674516

[B30] Chen J. Gao C. Luo M. Zheng C. Lin X. Ning Y. (2022). MicroRNA‐202 safeguards meiotic progression by preventing premature SEPARASE‐mediated REC8 cleavage. EMBO Rep. 23, e54298. 10.15252/EMBR.202154298 35712867 PMC9346496

[B31] Chengizkhan G. Bhaskaran N. Ileng Kumaran R. Ramachandran I. (2020). Cancer stem cells and tumour aggressiveness. Cancer Stem Cells New Horizons Cancer Ther., 137–154. 10.1007/978-981-15-5120-8_8

[B32] Cheung K. J. Ewald A. J. (2016). A collective route to metastasis: seeding by tumor cell clusters. Science 352, 167–169. 10.1126/SCIENCE.AAF6546 27124449 PMC8183671

[B33] Chew G. L. Campbell A. E. De Neef E. Sutliff N. A. Shadle S. C. Tapscott S. J. (2019). DUX4 suppresses MHC class I to promote cancer immune evasion and resistance to checkpoint blockade. Dev. Cell 50, 658–671. 10.1016/J.DEVCEL.2019.06.011 31327741 PMC6736738

[B34] Choi Y. J. Lin C. P. Ho J. J. He X. Okada N. Bu P. (2011). miR-34 miRNAs provide a barrier for somatic cell reprogramming. Nat. Cell Biol. 13, 1353–1360. 10.1038/NCB2366 22020437 PMC3541684

[B35] Christodoulou F. Raible F. Tomer R. Simakov O. Trachana K. Klaus S. (2010). Ancient animal microRNAs and the evolution of tissue identity. Nature 463, 1084–1088. 10.1038/NATURE08744 20118916 PMC2981144

[B36] Chuai M. Hughes D. J. Weijer C. (2012). Collective epithelial and mesenchymal cell migration during gastrulation. Curr. Genomics 13, 267–277. 10.2174/138920212800793357 23204916 PMC3394114

[B37] Cioffi M. Trabulo S. M. Sanchez-Ripoll Y. Miranda-Lorenzo I. Lonardo E. Dorado J. (2015). The miR-17-92 cluster counteracts quiescence and chemoresistance in a distinct subpopulation of pancreatic cancer stem cells. Gut 64, 1936–1948. 10.1136/GUTJNL-2014-308470/-/DC1 25887381 PMC4680182

[B38] Cismaru C. A. Pop L. Berindan-Neagoe I. (2018). Incognito: are microchimeric fetal stem cells that cross placental barrier real emissaries of peace? Stem Cell Rev. Rep. 14, 632–641. 10.1007/S12015-018-9834-9 29948753

[B39] Cismaru C. A. Soritau O. Jurj A.-M. Lajos R. Pop B. Bocean C. (2019). Isolation and characterization of a fetal-maternal microchimeric stem cell population in maternal hair follicles long after parturition. Stem Cell Rev. Rep. 15, 519–529. 10.1007/S12015-019-09885-4 31123983

[B40] Cismaru A. C. Soritau O. Jurj A. M. Raduly L.-Z. Pop B. Bocean C. (2020). Human chorionic gonadotropin improves the proliferation and regenerative potential of bone marrow adherent stem cells and the immune tolerance of fetal microchimeric stem cells *in vitro* . Stem Cell Rev. Rep. 163 (16), 524–540. 10.1007/S12015-020-09957-W 32020407

[B41] Cismaru C. A. Pirlog R. Calin G. A. Berindan-Neagoe I. (2022). Stem cells in the tumor immune microenvironment –Part of the cure or part of the disease? Ontogeny and dichotomy of stem and immune cells has led to better understanding. Stem Cell Rev. Rep. 1, 1–17. 10.1007/S12015-022-10428-7 35841518

[B42] Cismaru C. A. Tomuleasa C. Jurj A. Chira S. Isachekcu E. Cismaru G. (2024). Synergistic effect of human chorionic gonadotropin and granulocyte colony stimulating factor in the mobilization of HSPCs improves overall survival after PBSCT in a preclinical murine model. Are we far enough for therapy? Stem Cell Rev. Reports 20, 206–217. 10.1007/S12015-023-10648-5 37922107

[B43] Clarke M. F. Dick J. E. Dirks P. B. Eaves C. J. Jamieson C. H. M. Jones D. L. (2006). Cancer stem cells - perspectives on current status and future directions: AACR workshop on cancer stem cells. Cancer Res. 66, 9339–9344. 10.1158/0008-5472.CAN-06-3126 16990346

[B44] Collu G. M. Hidalgo-Sastre A. Brennan K. (2014). Wnt–notch signalling crosstalk in development and disease. Cell. Mol. Life Sci. C 71, 3553–3567. 10.1007/S00018-014-1644-X 24942883 PMC11113451

[B45] Cook M. S. Munger S. C. Nadeau J. H. Cape B. (2011). Regulation of Male germ cell cycle arrest and differentiation by DND1 is modulated by genetic background. Development 138, 23–32. 10.1242/DEV.057000 21115610 PMC2998163

[B46] Coorens T. H. H. Oliver T. R. W. Sanghvi R. Sovio U. Cook E. Vento-Tormo R. (2021). Inherent mosaicism and extensive mutation of human placentas. Nat 2021, 80–85. 10.1038/s41586-021-03345-1 33692543 PMC7611644

[B47] Coufal N. G. Garcia-Perez J. L. Peng G. E. Yeo G. W. Mu Y. Lovci M. T. (2009). L1 retrotransposition in human neural progenitor cells. Nature 460, 1127–1131. 10.1038/NATURE08248 19657334 PMC2909034

[B48] Crowell R. C. Kiessling A. A. (2007). Endogenous retrovirus expression in testis and epididymis. Biochem. Soc. Trans. 35, 629–633. 10.1042/BST0350629 17511667

[B49] Cubela S. (2008). SPATA2 is downregulated during pancreatic development and is a target of mir-7, a pancreatic microRNA predominantly expressed in islets. Available online at: https://www.academia.edu/93095900/SPATA2_is_downregulated_during_pancreatic_development_and_is_a_target_of_mir_7_a_pancreatic_microRNA_predominantly_expressed_in_islets (Accessed June 27, 2025).

[B50] Cusulin C. Monni E. Ahlenius H. Wood J. Brune J. C. Lindvall O. (2012). Embryonic stem cell-derived neural stem cells fuse with microglia and mature neurons. Stem Cells 30, 2657–2671. 10.1002/STEM.1227 22961761

[B51] Dadoune J. P. Pawlak A. Alfonsi M. F. Siffroi J. P. (2005). Identification of transcripts by macroarrays, RT-PCR and *in situ* hybridization in human ejaculate spermatozoa. Mol. Hum. Reprod. 11, 133–140. 10.1093/MOLEHR/GAH137 15591450

[B52] Dainiak N. Hoffman R. (1980). Hemoglobin F production in testicular malignancy. Cancer 45, 2177–2180. 10.1002/1097-0142(19800415)45:8<2177::aid-cncr2820450828>3.0.co;2-w 6154526

[B53] Daskalos A. Nikolaidis G. Xinarianos G. Savvari P. Cassidy A. Zakopoulou R. (2009). Hypomethylation of retrotransposable elements correlates with genomic instability in non-small cell lung cancer. Int. J. Cancer 124, 81–87. 10.1002/IJC.23849 18823011

[B54] Defossez P.-A. Gupta N. Yakhou L. Albert J. R. Miura F. Ferry L. (2022). A genome-wide screen reveals new regulators of the 2-cell-like cell state. 10.21203/RS.3.RS-1561018/V1 37488355

[B55] Deniz Ö. Frost J. M. Branco M. R. (2019). Regulation of transposable elements by DNA modifications. Nat. Rev. Genet. 20, 417–431. 10.1038/S41576-019-0106-6 30867571

[B56] Dermitzakis I. Manthou M. E. Meditskou S. Tremblay M. È. Petratos S. Zoupi L. (2023). Origin and emergence of microglia in the CNS—An interesting (Hi)story of an eccentric cell. Curr. Issues Mol. Biol. 45, 2609–2628. 10.3390/CIMB45030171 36975541 PMC10047736

[B57] DeSouza N. R. Carnazza M. Jarboe T. Quaranto D. Kopec K. Centone A. J. (2025). Long non-coding RNA DUXAP10 promotes tumorigenesis and metastasis in Anaplastic thyroid cancer. Cancers 17, 3852. 10.3390/CANCERS17233852 41375055 PMC12691012

[B58] Dittmar T. Nagler C. Schwitalla S. Reith G. Niggemann B. Zänker K. S. (2009). Recurrence cancer stem cells--made by cell fusion? Med. Hypotheses 73, 542–547. 10.1016/J.MEHY.2009.05.044 19564079

[B59] Dockree S. Shine B. Pavord S. Impey L. Vatish M. (2021). White blood cells in pregnancy: reference intervals for before and after delivery. EBioMedicine 74, 103715. 10.1016/j.ebiom.2021.103715 34826802 PMC8626574

[B60] Doitsidou M. Reichman-Fried M. Stebler J. Köprunner M. Dörries J. Meyer D. (2002). Guidance of primordial germ cell migration by the chemokine SDF-1. Cell 111, 647–659. 10.1016/S0092-8674(02)01135-2 12464177

[B61] Durcova-Hills G. Ainscough J. F. X. McLaren A. (2001). Pluripotential stem cells derived from migrating primordial germ cells. Differentiation 68, 220–226. 10.1046/j.1432-0436.2001.680409.x 11776474

[B62] D’Alessandre N. D. R. Pessoa B. S. Guardia G. D. A. Marques J. M. Galante P. A. F. Batista R. L. (2025). Retroelements in thyroid cancer: epigenetic plasticity, dedifferentiation, and therapeutic opportunities. Rev. Endocr. Metab. Disord. 2025, 1–13. 10.1007/S11154-025-10008-3 41296188

[B63] Falick Michaeli T. Bergman Y. Gielchinsky Y. (2015). Rejuvenating effect of pregnancy on the mother. Fertil. Steril. 103, 1125–1128. 10.1016/j.fertnstert.2015.02.034 25813291

[B64] Ferrand J. Noël D. Lehours P. Prochazkova-Carlotti M. Chambonnier L. Ménard A. (2011). Human bone marrow-derived stem cells acquire epithelial characteristics through fusion with gastrointestinal epithelial cells. PLoS One 6, e19569. 10.1371/JOURNAL.PONE.0019569 21573181 PMC3088703

[B65] Ficz G. Reik W. (2013). Reprogramming by cell fusion: boosted by tets. Mol. Cell 49, 1017–1018. 10.1016/J.MOLCEL.2013.03.014 23541036

[B66] Garg M. (2017). Epithelial plasticity and cancer stem cells: major mechanisms of cancer pathogenesis and therapy resistance. World J. Stem Cells 9, 118–126. 10.4252/wjsc.v9.i8.118 28928908 PMC5583530

[B67] Geck P. (2013). Symptotic detection of chimerism: y does it matter? Chimerism 4, 144–146. 10.4161/CHIM.27095 24241283 PMC3921198

[B68] Geijsen N. Daley G. Q. (2008). Modeling germ cell differentiation. StemBook. 10.3824/stembook.1.29.1 20614586

[B69] Georgiou I. Noutsopoulos D. Dimitriadou E. Markopoulos G. Apergi A. Lazaros L. (2009). Retrotransposon RNA expression and evidence for retrotransposition events in human oocytes. Hum. Mol. Genet. 18, 1221–1228. 10.1093/HMG/DDP022 19147684

[B70] Giglia-Mari G. Zotter A. Vermeulen W. (2011). DNA damage response. Cold Spring Harb. Perspect. Biol. 3, a000745. 10.1101/cshperspect.a000745 20980439 PMC3003462

[B71] Gkountela S. Castro-Giner F. Szczerba B. M. Vetter M. Landin J. Scherrer R. (2019). Circulating tumor cell clustering shapes DNA methylation to enable metastasis seeding. Cell 176, 98–112. 10.1016/J.CELL.2018.11.046 30633912 PMC6363966

[B72] Glass R. Asirvatham J. R. Kahn L. Aziz M. (2015). Beta-human chorionic gonadotropin producing osteosarcoma of the sacrum in a 26-Year-Old woman: a case report and review of the literature. Case Rep. Pathol. 2015, 897230. 10.1155/2015/897230 25722909 PMC4334429

[B73] Gomzikova M. O. James V. Rizvanov A. A. (2021). Mitochondria donation by mesenchymal stem cells: current understanding and mitochondria transplantation strategies. Front. Cell Dev. Biol. 9, 653322. 10.3389/FCELL.2021.653322/BIBTEX 33898449 PMC8058353

[B74] Govatati S. Kodati V. L. Deenadayal M. Chakravarty B. Shivaji S. Bhanoori M. (2014). Mutations in the PTEN tumor gene and risk of endometriosis: a case-control study. Hum. Reprod. 29, 324–336. 10.1093/HUMREP/DET387 24154570

[B75] Grelet S. Andries V. Polette M. Gilles C. Staes K. Martin A. P. (2015). The human NANOS3 gene contributes to lung tumour invasion by inducing epithelial-mesenchymal transition. J. Pathol. 237, 25–37. 10.1002/PATH.4549 25904364

[B76] Grundy E. Tomassini C. (2005). Fertility history and health in later life: a record linkage study in England and Wales. Soc. Sci. Med. 61, 217–228. 10.1016/j.socscimed.2004.11.046 15847974

[B77] Hackett J. A. Sengupta R. Zylicz J. J. Murakami K. Lee C. Down T. A. (2012). Germline DNA demethylation dynamics and imprint erasure through 5-hydroxymethylcytosine. Science 339, 448–452. 10.1126/SCIENCE.1229277 23223451 PMC3847602

[B78] Haggerty C. Kretzmer H. Riemenschneider C. Kumar A. S. Mattei A. L. Bailly N. (2021). Dnmt1 has *de novo* activity targeted to transposable elements. Nat. Struct. Mol. Biol. 28, 594–603. 10.1038/S41594-021-00603-8 34140676 PMC8279952

[B79] Halaban R. Nordlund J. Francke U. Moellmann G. Eisenstadt J. M. (1980). Supermelanotic hybrids derived from mouse melanomas and normal mouse cells. Somat. Cell Genet. 6, 29–44. 10.1007/BF01538694 6768142

[B80] Halik P. K. Koźmiński P. Gniazdowska E. (2020). Perspectives of methotrexate-based radioagents for application in nuclear medicine. Mol. Pharm. 18, 33. 10.1021/ACS.MOLPHARMACEUT.0C00740 33251808 PMC7788572

[B81] Hanahan D. (2022). Hallmarks of cancer: new DimensionsHallmarks of cancer: new dimensions. Cancer Discov. 12, 31–46. 10.1158/2159-8290.CD-21-1059 35022204

[B82] Handschuh K. Guibourdenche J. Tsatsaris V. Guesnon M. Laurendeau I. Evain-Brion D. (2007). Human chorionic gonadotropin produced by the invasive trophoblast but not the villous trophoblast promotes cell invasion and is down-regulated by peroxisome proliferator-activated receptor-gamma. Endocrinology 148, 5011–5019. 10.1210/EN.2007-0286 17628005

[B83] Hashimoto D. Chow A. Noizat C. Teo P. Beasley M. B. Leboeuf M. (2013). Tissue-resident macrophages self-maintain locally throughout adult life with minimal contribution from circulating monocytes. Immunity 38, 792–804. 10.1016/J.IMMUNI.2013.04.004 23601688 PMC3853406

[B84] Heaney J. D. Anderson E. L. Michelson M. V. Zechel J. L. Conrad P. A. Page D. C. (2012). Germ cell pluripotency, premature differentiation and susceptibility to testicular teratomas in mice. Development 139, 1577–1586. 10.1242/DEV.076851 22438569 PMC3317965

[B85] Hendrickson P. G. Doráis J. A. Grow E. J. Whiddon J. L. Lim J. W. Wike C. L. (2017). Conserved roles of mouse DUX and human DUX4 in activating cleavage-stage genes and MERVL/HERVL retrotransposons. Nat. Genet. 49, 925–934. 10.1038/NG.3844 28459457 PMC5703070

[B86] Hollister J. D. Gaut B. S. (2009). Epigenetic silencing of transposable elements: a trade-off between reduced transposition and deleterious effects on neighboring gene expression. Genome Res. 19, 1419–1428. 10.1101/GR.091678.109 19478138 PMC2720190

[B87] Houbaviy H. B. Murray M. F. Sharp P. A. (2003). Embryonic stem cell-specific MicroRNAs. Dev. Cell 5, 351–358. 10.1016/S1534-5807(03)00227-2 12919684

[B88] Howell J. C. Lee W. H. Morrison P. Zhong J. Yoder M. C. Srour E. F. (2003). Pluripotent stem cells identified in multiple murine tissues. Ann. N. Y. Acad. Sci. 996, 158–173. 10.1111/J.1749-6632.2003.TB03244.X;ISSUE:ISSUE 12799294

[B89] Hu J. Zhao Q. Feng Y. Li N. Gu Y. Sun R. (2018). Embryonic germ cell extracts erase imprinted genes and improve the efficiency of induced pluripotent stem cells. Sci. Rep. 2018 8, 10955. 10.1038/s41598-018-29339-0 30026469 PMC6053380

[B90] Huang Y. Hong W. Wei X. (2022). The molecular mechanisms and therapeutic strategies of EMT in tumor progression and metastasis. J. Hematol. Oncol. 15, 129. 10.1186/S13045-022-01347-8 36076302 PMC9461252

[B91] Huyhn A. Dommergues M. Izac B. Croisille L. Katz A. Vainchenker W. (1995). Characterization of hematopoietic progenitors from human yolk sacs and embryos. Blood 86, 4474–4485. 10.1182/BLOOD.V86.12.4474.BLOODJOURNAL86124474 8541536

[B92] Hyde J. R. Kamangar E. Ladenheim A. E. Cooke D. T. (2023). Benign lung adenoma mimicking an adenocarcinoma with EML4-ALK gene fusion. Ann. Thorac. Surg. Short. Rep. 1, 137–139. 10.1016/J.ATSSR.2022.10.006 39790518 PMC11708699

[B93] Iles R. K. Delves P. J. Butler S. A. (2010). Does hCG or hCGβ play a role in cancer cell biology? Mol. Cell. Endocrinol. 329, 62–70. 10.1016/J.MCE.2010.07.014 20654692

[B94] Inst E. W.-C. E. C. (1948). Migration of the germ cells of human embryos from the yolk sac to the primitive gonadal folds. Available online at: https://cir.nii.ac.jp/crid/1570854174598416384 (Accessed January 10, 2025).

[B95] Iorio F. Garcia-Alonso L. Brammeld J. S. Martincorena I. Wille D. R. McDermott U. (2018). Pathway-based dissection of the genomic heterogeneity of cancer hallmarks’ acquisition with SLAPenrich. Sci. Rep. 8, 6713. 10.1038/S41598-018-25076-6 29713020 PMC5928049

[B96] Irie N. Weinberger L. Tang W. W. C. Kobayashi T. Viukov S. Manor Y. S. (2015). SOX17 is a critical specifier of human primordial germ cell fate. Cell 160, 253–268. 10.1016/J.CELL.2014.12.013 25543152 PMC4310934

[B97] Jachowicz J. W. Bing X. Pontabry J. Bošković A. Rando O. J. Torres-Padilla M. E. (2017). LINE-1 activation after fertilization regulates global chromatin accessibility in the early mouse embryo. Nat. Genet. 49, 1502–1510. 10.1038/ng.3945 28846101

[B98] Jang H. S. Shah N. M. Du A. Y. Dailey Z. Z. Pehrsson E. C. Godoy P. M. (2019). Transposable elements drive widespread expression of oncogenes in human cancers. Nat. Genet. 51, 611–617. 10.1038/s41588-019-0373-3 30926969 PMC6443099

[B99] Janowski M. (2009). Functional diversity of SDF-1 splicing variants. Cell adh. Migr. 3, 243–249. 10.4161/CAM.3.3.8260 19287206 PMC2712802

[B100] Kalluri R. Weinberg R. A. (2009). The basics of epithelial-mesenchymal transition. J. Clin. Invest. 119, 1420–1428. 10.1172/JCI39104 19487818 PMC2689101

[B101] Kamata T. Sunami K. Yoshida A. Shiraishi K. Furuta K. Shimada Y. (2016). Frequent BRAF or EGFR mutations in ciliated muconodular papillary tumors of the lung. J. Thorac. Oncol. 11, 261–265. 10.1016/J.JTHO.2015.10.021 26718882

[B102] Karlsson M. Zhang C. Méar L. Zhong W. Digre A. Katona B. (2021). A single–cell type transcriptomics map of human tissues. Sci. Adv. 7, eabh2169. 10.1126/SCIADV.ABH2169;CTYPE:STRING:JOURNAL 34321199 PMC8318366

[B103] Kerbel R. S. Lagarde A. E. Dennis J. W. Donaghue T. P. (1983). Spontaneous fusion *in vivo* between normal host and tumor cells: possible contribution to tumor progression and metastasis studied with a lectin-resistant mutant tumor. Mol. Cell. Biol. 3, 523–538. 10.1128/mcb.3.4.523 6687920 PMC368568

[B104] Kester L. van Oudenaarden A. (2018). Single-cell transcriptomics meets lineage tracing. Cell Stem Cell 23, 166–179. 10.1016/j.stem.2018.04.014 29754780

[B105] Kim C. H. (2010). Homeostatic and pathogenic extramedullary hematopoiesis. J. Blood Med. 1, 13–19. 10.2147/JBM.S7224 22282679 PMC3262334

[B106] Kim F. J. Battini J. L. Manel N. Sitbon M. (2004). Emergence of vertebrate retroviruses and envelope capture. Virology 318, 183–191. 10.1016/J.VIROL.2003.09.026 14972546

[B107] Kim Y. S. Yi B. R. Kim N. H. Choi K. C. (2014). Role of the epithelial–mesenchymal transition and its effects on embryonic stem cells. Exp. Mol. Med. 46, e108. 10.1038/emm.2014.44 25081188 PMC4150931

[B108] Köhler G. Milstein C. (1975). Continuous cultures of fused cells secreting antibody of predefined specificity. Nature 256, 495–497. 10.1038/256495A0 1172191

[B109] Krawetz S. A. Kruger A. Lalancette C. Tagett R. Anton E. Draghici S. (2011). A survey of small RNAs in human sperm. Hum. Reprod. 26, 3401–3412. 10.1093/HUMREP/DER329 21989093 PMC3212879

[B110] Krisher R. L. Prather R. S. (2012). A role for the warburg effect in preimplantation embryo development: Metabolic modification to support rapid cell proliferation. Mol. Reprod. Dev. 79, 311–320. 10.1002/MRD.22037 22431437 PMC3328638

[B111] Küppers R. Hansmann M. L. (2005). The hodgkin and reed/sternberg cell. Int. J. Biochem. Cell Biol. 37, 511–517. 10.1016/J.BIOCEL.2003.10.025 15618006

[B112] Lacham-Kaplan O. Daniels R. Trounson A. (2001). Fertilization of mouse oocytes using somatic cells as male germ cells. Reprod. Biomed. Online 3, 205–211. 10.1016/S1472-6483(10)62037-8 12513856

[B113] Lanza D. G. Dawson E. P. Rao P. Heaney J. D. (2016). Misexpression of cyclin D1 in embryonic germ cells promotes testicular teratoma initiation. Cell Cycle 15, 919–930. 10.1080/15384101.2016.1149272 26901436 PMC4889263

[B114] Laurent L. C. (2008). MicroRNAs in embryonic stem cells and early embryonic development. J. Cell. Mol. Med. 12, 2181–2188. 10.1111/J.1582-4934.2008.00513.X 19120702 PMC4514098

[B115] Law P. J. Timofeeva M. Fernandez-Rozadilla C. Broderick P. Studd J. Fernandez-Tajes J. (2019). Association analyses identify 31 new risk loci for colorectal cancer susceptibility. Nat. Commun. 10, 2154. 10.1038/S41467-019-09775-W 31089142 PMC6517433

[B116] Lehka L. Rędowicz M. J. (2020). Mechanisms regulating myoblast fusion: a multilevel interplay. Semin. Cell Dev. Biol. 104, 81–92. 10.1016/J.SEMCDB.2020.02.004 32063453

[B117] Leroy H. Han M. Woottum M. Bracq L. Bouchet J. Xie M. (2020). Virus-mediated cell-cell fusion. Int. J. Mol. Sci. 21, 1–28. 10.3390/IJMS21249644 33348900 PMC7767094

[B118] Lesch B. J. Page D. C. (2012). Genetics of germ cell development. Nat. Rev. Genet. 13, 781–794. 10.1038/nrg3294 23044825

[B119] Levaot N. Ottolenghi A. Mann M. Guterman-Ram G. Kam Z. Geiger B. (2015). Osteoclast fusion is initiated by a small subset of RANKL-stimulated monocyte progenitors, which can fuse to RANKL-unstimulated progenitors. Bone 79, 21–28. 10.1016/j.bone.2015.05.021 26008608

[B120] Lin A. Yan W. H. (2015). Human leukocyte Antigen-G (HLA-G) expression in cancers: roles in immune evasion, metastasis and target for therapy. Mol. Med. 21, 782–791. 10.2119/MOLMED.2015.00083 26322846 PMC4749493

[B121] Lin Y. H. Zhang S. Zhu M. Lu T. Chen K. Wen Z. (2020). Mice with increased numbers of polyploid hepatocytes maintain regenerative capacity but develop fewer hepatocellular carcinomas following chronic liver injury. Gastroenterology 158, 1698–1712. 10.1053/J.GASTRO.2020.01.026 31972235 PMC8902703

[B122] Lin L. Li Q. Wang Y. Shi Y. (2021). Syncytia formation during SARS-CoV-2 lung infection: a disastrous unity to eliminate lymphocytes. Cell Death Differ. 28, 2019–2021. 10.1038/s41418-021-00795-y 33981020 PMC8114657

[B123] Lindehell H. Schwartz Y. B. Larsson J. (2023). Methylation of lysine 36 on histone H3 is required to control transposon activities in somatic cells. Life Sci. Alliance 6, e202201832. 10.26508/LSA.202201832 37169594 PMC10176111

[B124] Lingg L. Rottenberg S. Francica P. (2022). Meiotic genes and DNA double strand break repair in cancer. Front. Genet. 13, 831620. 10.3389/FGENE.2022.831620/BIBTEX 35251135 PMC8895043

[B125] Liu J. (2022). Giant cells: linking McClintock’s heredity to early embryogenesis and tumor origin throughout millennia of evolution on Earth. Semin. Cancer Biol. 81, 176–192. 10.1016/j.semcancer.2021.06.007 34116161

[B126] Liu C. Moten A. Ma Z. Lin H. K. (2022a). The foundational framework of tumors: gametogenesis, p53, and cancer. Semin. Cancer Biol. 81, 193–205. 10.1016/j.semcancer.2021.04.018 33940178 PMC9382687

[B127] Liu Y. Lu X. Ye M. Wang L. Tang R. Yang Z. (2022b). Efficient silencing of the multicopy DUX4 gene by ABE-mediated start codon mutation in human embryos. J. Genet. Genomics 49, 982–985. 10.1016/J.JGG.2022.02.010 35231637

[B128] Lluis F. Cosma M. P. (2010). Cell-fusion-mediated somatic-cell reprogramming: a mechanism for tissue regeneration. J. Cell. Physiol. 223, 6–13. 10.1002/jcp.22003 20049847

[B129] Loke Y. W. King A. Chumbley G. (1994). Human trophoblast cell surface molecules: HLA-G and reproduction: a review. Placenta 15, 331–337. 10.1016/S0143-4004(05)80355-8

[B130] Lomax M. E. Barnes D. M. Hupp T. R. Picksley S. M. Camplejohn R. S. (1998). Characterization of p53 oligomerization domain mutations isolated from Li-Fraumeni and Li-Fraumeni like family members. Oncogene 17, 643–649. 10.1038/SJ.ONC.1201974 9704930

[B131] Lu L. Wang J. R. Henderson Y. C. Bai S. Yang J. Hu M. (2023). Anaplastic transformation in thyroid cancer revealed by single-cell transcriptomics. J. Clin. Invest. 133, e169653. 10.1172/JCI169653 37053016 PMC10231997

[B132] Lugassy C. Zadran S. Bentolila L. A. Wadehra M. Prakash R. Thomas Carmichael S. (2014). Angiotropism, pericytic mimicry and extravascular migratory metastasis in melanoma: an alternative to intravascular cancer dissemination. Cancer Microenviron. 7, 139–152. 10.1007/s12307-014-0156-4 25304454 PMC4275501

[B133] Lynch-Sutherland C. F. Chatterjee A. Stockwell P. A. Eccles M. R. Macaulay E. C. (2020). Reawakening the developmental origins of cancer through transposable elements. Front. Oncol. 10, 468. 10.3389/FONC.2020.00468 32432029 PMC7214541

[B134] Ma Y. Zhang P. Wang F. Yang J. Yang Z. Qin H. (2010). The relationship between early embryo development and tumourigenesis. J. Cell. Mol. Med. 14, 2697–2701. 10.1111/J.1582-4934.2010.01191.X 21029369 PMC3822720

[B135] Maggio M. Snyder P. J. Ceda G. P. Milaneschi Y. Luci M. Cattabiani C. (2013). Is the haematopoietic effect of testosterone mediated by erythropoietin? The results of a clinical trial in older men. Andrology 1, 24–28. 10.1111/J.2047-2927.2012.00009.X 23258626

[B136] Manaster I. Goldman-Wohl D. Greenfield C. Nachmani D. Tsukerman P. Hamani Y. (2012). MiRNA-mediated control of HLA-G expression and function. PLoS One 7, e33395. 10.1371/JOURNAL.PONE.0033395 22438923 PMC3306401

[B137] Maroldi R. Farina D. Borghesi A. Marconi A. Gatti E. (2008). Perineural tumor spread. Neuroimaging Clin. N. Am. 18, 413–429. 10.1016/J.NIC.2008.01.001 18466839

[B138] Martincorena I. Fowler J. C. Wabik A. Lawson A. R. J. Abascal F. Hall M. W. J. (2018). Somatic mutant clones colonize the human esophagus with age. Science 362, 911–917. 10.1126/science.aau3879 30337457 PMC6298579

[B139] Marusyk A. Polyak K. (2010). Tumor heterogeneity: causes and consequences. Biochim. Biophys. Acta 1805, 105–117. 10.1016/J.BBCAN.2009.11.002 19931353 PMC2814927

[B140] Matsumoto T. Wakefield L. Peters A. Peto M. Spellman P. Grompe M. (2021). Proliferative polyploid cells give rise to tumors *via* ploidy reduction. Nat. Commun. 12, 646. 10.1038/S41467-021-20916-Y 33510149 PMC7843634

[B141] McClatchey S. T. H. Wang Z. Linden L. M. Hastie E. L. Wang L. Shen W. (2016). Boundary cells restrict dystroglycan trafficking to control basement membrane sliding during tissue remodeling. Elife 5. 10.7554/ELIFE.17218 27661254 PMC5061546

[B142] McFarlane R. J. Wakeman J. A. (2017). Meiosis-like functions in oncogenesis: a new view of cancer. Cancer Res. 77, 5712–5716. 10.1158/0008-5472.CAN-17-1535 29061671

[B143] Mendell J. T. (2008). miRiad roles for the miR-17-92 cluster in development and disease. Cell 133, 217–222. 10.1016/J.CELL.2008.04.001 18423194 PMC2732113

[B144] Mendez-Dorantes C. Burns K. H. (2023). LINE-1 retrotransposition and its deregulation in cancers: implications for therapeutic opportunities. Genes Dev. 37, 948–967. 10.1101/GAD.351051.123 38092519 PMC10760644

[B145] Mendt M. Cardier J. E. (2015). Role of SDF-1 (CXCL12) in regulating hematopoietic stem and progenitor cells traffic into the liver during extramedullary hematopoiesis induced by G-CSF, AMD3100 and PHZ. Cytokine 76, 214–221. 10.1016/J.CYTO.2015.05.004 26093947

[B146] Micalizzi D. S. Farabaugh S. M. Ford H. L. (2010). Epithelial-mesenchymal transition in cancer: parallels between normal development and tumor progression. J. Mammary Gland. Biol. Neoplasia 15, 117–134. 10.1007/S10911-010-9178-9/FIGURES/6 20490631 PMC2886089

[B147] Mierzejewska K. Borkowska S. Suszynska E. Suszynska M. Poniewierska-Baran A. Maj M. (2015). Hematopoietic stem/progenitor cells express several functional sex hormone receptors—novel evidence for a potential developmental link between hematopoiesis and primordial germ cells. Stem Cells Dev. 24, 927–937. 10.1089/SCD.2014.0546 25607657 PMC4390002

[B148] Miller F. R. McInerney D. Rogers C. Miller B. E. (1988). Spontaneous fusion between metastatic mammary tumor subpopulations. J. Cell. Biochem. 36, 129–136. 10.1002/jcb.240360204 3356752

[B149] Molinaro E. Romei C. Biagini A. Sabini E. Agate L. Mazzeo S. (2017). Anaplastic thyroid carcinoma: from clinicopathology to genetics and advanced therapies. Nat. Rev. Endocrinol. 13, 644–660. 10.1038/NRENDO.2017.76 28707679

[B150] Møllgård K. Jespersen A. Lutterodt M. C. Yding Andersen C. Høyer P. E. Byskov A. G. (2010). Human primordial germ cells migrate along nerve fibers and schwann cells from the dorsal hind gut mesentery to the gonadal ridge. Mol. Hum. Reprod. 16, 621–631. 10.1093/MOLEHR/GAQ052 20566702

[B151] Montalto F. I. De Amicis F. (2020). Cyclin D1 in cancer: a molecular connection for cell cycle control, adhesion and invasion in tumor and stroma. Cells 9, 2648. 10.3390/CELLS9122648 33317149 PMC7763888

[B152] Moore L. B. Sawyer A. J. Saucier-Sawyer J. Saltzman W. M. Kyriakides T. R. (2016). Nanoparticle delivery of miR-223 to attenuate macrophage fusion. Biomaterials 89, 127–135. 10.1016/J.BIOMATERIALS.2016.02.036 26967647 PMC4924476

[B153] Morales-Prieto D. M. Chaiwangyen W. Ospina-Prieto S. Schneider U. Herrmann J. Gruhn B. (2012). MicroRNA expression profiles of trophoblastic cells. Placenta 33, 725–734. 10.1016/j.placenta.2012.05.009 22721760

[B154] Muñoz-Espín D. Serrano M. (2014). Cellular senescence: from physiology to pathology. Nat. Rev. Mol. Cell Biol. 15, 482–496. 10.1038/nrm3823 24954210

[B155] Muotri A. R. Chu V. T. Marchetto M. C. N. Deng W. Moran J. V. Gage F. H. (2005). Somatic mosaicism in neuronal precursor cells mediated by L1 retrotransposition. Nature 435, 903–910. 10.1038/NATURE03663 15959507

[B156] Nagamatsu G. Kosaka T. Kawasumi M. Kinoshita T. Takubo K. Akiyama H. (2011). A germ cell-specific gene, Prmt5, works in somatic cell reprogramming. J. Biol. Chem. 286, 10641–10648. 10.1074/JBC.M110.216390 21270127 PMC3060515

[B157] Nagamatsu G. Kosaka T. Saito S. Honda H. Takubo K. Kinoshita T. (2013). Induction of pluripotent stem cells from primordial germ cells by single reprogramming factors. Stem Cells 31, 479–487. 10.1002/STEM.1303 23255173

[B158] Nam C. H. Youk J. Kim J. Y. Lim J. Park J. W. Oh S. A. (2023). Widespread somatic L1 retrotransposition in normal colorectal epithelium. Nature 617, 540–547. 10.1038/S41586-023-06046-Z 37165195 PMC10191854

[B159] Naxerova K. Bult C. J. Peaston A. Fancher K. Knowles B. B. Kasif S. (2008). Analysis of gene expression in a developmental context emphasizes distinct biological leitmotifs in human cancers. Genome Biol. 9, R108. 10.1186/GB-2008-9-7-R108 18611264 PMC2530866

[B160] Negrini S. Gorgoulis V. G. Halazonetis T. D. (2010). Genomic instability an evolving hallmark of cancer. Nat. Rev. Mol. Cell Biol. 11, 220–228. 10.1038/NRM2858 20177397

[B161] Ng P. Cheng-I W. Ng P. Cheng-I W. (2013). The dark side of pluripotency – cancer stem cell. Pluripotent Stem Cells. 10.5772/54369

[B162] Ohms S. Lee S. H. Rangasamy D. (2014). LINE-1 retrotransposons and let-7 miRNA: partners in the pathogenesis of cancer? Front. Genet. 5, 338. 10.3389/FGENE.2014.00338/ABSTRACT 25339972 PMC4188135

[B163] Old L. J. (2007). Cancer is a somatic cell pregnancy. Cancer Immun. a J. Acad. Cancer Immunol. 7, 19. Available online at: https://pmc.ncbi.nlm.nih.gov/articles/PMC2935741/(Accessed February 13, 2026). 17983204 PMC2935741

[B164] Oliveira P. F. Martins A. D. Moreira A. C. Cheng C. Y. Alves M. G. (2014). The warburg effect revisited—lesson from the sertoli cell. Med. Res. Rev. 35, 126–151. 10.1002/MED.21325 25043918 PMC4845724

[B165] Oonakahara K. I. Matsuyama W. Higashimoto I. Kawabata M. Arimura K. Osame M. (2012). Stromal-derived Factor-1α/CXCL12-CXCR 4 axis is involved in the dissemination of NSCLC cells into pleural space. Am. J. Respir. Cell Mol. Biol. 30, 671–677. 10.1165/RCMB.2003-0340OC 14672915

[B166] Oosterhuis J. W. Looijenga L. H. J. (2019). Human germ cell tumours from a developmental perspective. Nat. Rev. Cancer 19, 522–537. 10.1038/S41568-019-0178-9 31413324

[B167] Pan J. Mestas J. Burdick M. D. Phillips R. J. Thomas G. V. Reckamp K. (2006). Stromal derived Factor-1 (SDF-1/CXCL12) and CXCR4 in renal cell carcinoma metastasis. Mol. Cancer 5, 1–14. 10.1186/1476-4598-5-56/TABLES/1 17083723 PMC1636662

[B168] Park J. W. Kwak J. Kim K.-W. Jung S. Nam C. H. Kim H. J. (2026). IDH-Mutant gliomas arise from glial progenitor cells harboring the initial driver mutation. Science 80, 391. 10.1126/SCIENCE.ADT0559 41505555

[B169] Pawelek J. M. (2000). Tumour cell hybridization and metastasis revisited. Melanoma Res. 10, 507–514. 10.1097/00008390-200012000-00001 11198471

[B170] Payer L. M. Burns K. H. (2019). Transposable elements in human genetic disease. Nat. Rev. Genet. 20, 760–772. 10.1038/S41576-019-0165-8 31515540

[B171] Pereda Tapiol J. Niimi G. (2008). Embryonic erythropoiesis in human yolk sac: two different compartments for two different processes. Microsc. Res. Tech. 71, 856–862. 10.1002/JEMT.20627 18767052

[B172] Pesaresi M. Sebastian-Perez R. Cosma M. P. (2019). Dedifferentiation, transdifferentiation and cell fusion: *in vivo* reprogramming strategies for regenerative medicine. FEBS J. 286, 1074–1093. 10.1111/FEBS.14633;JOURNAL:JOURNAL:14321033;ISSUE:ISSUE 30103260

[B173] Piccolo F. M. Bagci H. Brown K. E. Landeira D. Soza-Ried J. Feytout A. (2013). Different roles for Tet1 and Tet2 proteins in reprogramming-mediated erasure of imprints induced by EGC fusion. Mol. Cell 49, 1023–1033. 10.1016/J.MOLCEL.2013.01.032 23453809 PMC3613797

[B174] Pirlog R. Chiroi P. Raduly L. Nutu A. Cismaru A. Berindan-Neagoe I. (2023). Epithelial to mesenchymal transition in lung cancer: when it starts? 41–62. 10.1007/16833_2023_137

[B175] Posey A. D. Demonbreun A. McNally E. M. (2011). Ferlin proteins in myoblast fusion and muscle growth. Curr. Top. Dev. Biol. 96, 203–230. 10.1016/B978-0-12-385940-2.00008-5 21621072 PMC4464798

[B176] Pötgens A. J. G. Drewlo S. Kokozidou M. Kaufmann P. (2004). Syncytin: the major regulator of trophoblast fusion? Recent developments and hypotheses on its action. Hum. Reprod. Update 10, 487–496. 10.1093/humupd/dmh039 15333590

[B177] Pruteanu D. P. Olteanu D. E. Cosnarovici R. Mihut E. Nagy V. (2020). Genetic predisposition in pediatric oncology. Med. Pharm. Rep. 93, 323–334. 10.15386/MPR-1576 33225257 PMC7664724

[B178] Rainier S. Feinberg A. P. (1994). Genomic imprinting, DNA methylation, and cancer. JNCI J. Natl. Cancer Inst. 86, 753–759. 10.1093/JNCI/86.10.753 8169973

[B179] Ratajczak M. Z. (2015). A novel view of the adult bone marrow stem cell hierarchy and stem cell trafficking. Leukemia 29, 776–782. 10.1038/LEU.2014.346 25486871 PMC4396402

[B180] Ratajczak M. Z. (2017). Why are hematopoietic stem cells so “sexy”? – on a search for developmental explanation. Leukemia 31, 1671–1677. 10.1038/LEU.2017.148 28502982 PMC5540746

[B181] Ratajczak M. Z. Suszynska M. (2016). Emerging strategies to enhance homing and engraftment of hematopoietic stem cells. Stem Cell Rev. Rep. 12, 121–128. 10.1007/S12015-015-9625-5/FIGURES/1 26400757 PMC4720694

[B182] Ratajczak M. Z. Machalinski B. Wojakowski W. Ratajczak J. Kucia M. (2007). A hypothesis for an embryonic origin of pluripotent Oct-4+ stem cells in adult bone marrow and other tissues. Leukemia 21, 860–867. 10.1038/sj.leu.2404630 17344915

[B183] Ratajczak M. Z. Zuba-Surma E. K. Wojakowski W. Ratajczak J. Kucia M. (2008). Bone marrow – home of versatile stem cells. Transfus. Med. Hemotherapy 35, 248–259. 10.1159/000125585 21547122 PMC3083292

[B184] Ratajczak M. Z. Shin D. M. Kucia M. (2009). Very small Embryonic/epiblast-like stem cells: a missing link to support the germ line hypothesis of cancer development? Am. J. Pathol. 174, 1985–1992. 10.2353/AJPATH.2009.081143 19406990 PMC2684162

[B185] Ratajczak M. Z. Shin D. M. Liu R. Mierzejewska K. Ratajczak J. Kucia M. (2012). Very small embryonic/epiblast-like stem cells (VSELs) and their potential role in aging and organ rejuvenation – an update and comparison to other primitive small stem cells isolated from adult tissues. Aging (Albany NY) 4, 235–246. 10.18632/AGING.100449 22498452 PMC3371759

[B186] Ratajczak M. Z. Tarnowski M. Borkowska S. Serwin K. (2013). The embryonic rest hypothesis of cancer development: 150 years later. Trends Stem Cell Prolif. Cancer Res., 51–63. 10.1007/978-94-007-6211-4_3

[B187] Ratajczak M. Z. Ratajczak J. Suszynska M. Miller D. M. Kucia M. Shin D. M. (2017). A novel view of the adult stem cell compartment from the perspective of a quiescent population of very small embryonic-like stem cells. Circ. Res. 120, 166–178. 10.1161/CIRCRESAHA.116.309362 28057792 PMC5221475

[B188] Ratajczak M. Z. Ratajczak J. Kucia M. (2019). Very small embryonic-like stem cells (VSELs). Circ. Res. 124, 208–210. 10.1161/CIRCRESAHA.118.314287 30653438 PMC6461217

[B189] Ren J. Shi J. Zhang G. Wang J. (2022). A retroperitoneal perivascular ectopic pregnancy case: diagnosis and possible lymphatic migration. J. Minim. Invasive Gynecol. 29, 1203–1207. 10.1016/j.jmig.2022.06.019 35764248

[B190] Rengstl B. Newrzela S. Heinrich T. Weiser C. Thalheimer F. B. Schmid F. (2013). Incomplete cytokinesis and re-fusion of small mononucleated hodgkin cells lead to giant multinucleated reed-sternberg cells. Proc. Natl. Acad. Sci. U. S. A. 110, 20729–20734. 10.1073/PNAS.1312509110/SUPPL_FILE/SM08.MOV 24302766 PMC3870723

[B191] Robinson W. P. Price E. M. (2015). The human placental methylome. Cold Spring Harb. Perspect. Med. 5, a023044. 10.1101/CSHPERSPECT.A023044 25722473 PMC4448590

[B192] Rodriguez-Terrones D. Torres-Padilla M. E. (2018). Nimble and ready to mingle: Transposon outbursts of early development. Trends Genet. 34, 806–820. 10.1016/J.TIG.2018.06.006 30057183

[B193] Rosa A. Brivanlou A. H. (2017). Role of MicroRNAs in zygotic genome activation: modulation of mRNA during embryogenesis. Methods Mol. Biol. 1605, 31–43. 10.1007/978-1-4939-6988-3_3 28456956

[B194] Rossant J. Tam P. P. L. (2022). Early human embryonic development: blastocyst formation to gastrulation. Dev. Cell 57, 152–165. 10.1016/J.DEVCEL.2021.12.022 35077679

[B195] Rouillon C. Depincé A. Chênais N. Le Bail P. Y. Labbé C. (2019). Somatic cell nuclear transfer in non-enucleated goldfish oocytes: understanding DNA fate during oocyte activation and first cellular division. Sci. Rep. 91 (9), 1–12. 10.1038/s41598-019-48096-2 31462687 PMC6713701

[B196] Rueda A. Serna N. Mangues R. Villaverde A. Unzueta U. (2025). Targeting the chemokine receptor CXCR4 for cancer therapies. Biomark. Res. 13, 68. 10.1186/s40364-025-00778-y 40307933 PMC12044942

[B197] Ruvinsky A. (1999). Basics of gametic imprinting. J. Anim. Sci. 77, 228–237. 10.2527/1999.77SUPPL_2228X 15526800

[B198] Santarpia L. El-Naggar A. K. Cote G. J. Myers J. N. Sherman S. I. (2008). Phosphatidylinositol 3-kinase/akt and ras/raf-mitogen-activated protein kinase pathway mutations in anaplastic thyroid cancer. J. Clin. Endocrinol. Metab. 93, 278–284. 10.1210/JC.2007-1076 17989125

[B199] Sapir A. Avinoam O. Podbilewicz B. Chernomordik L. V. (2008). Viral and developmental cell fusion mechanisms: conservation and divergence. Dev. Cell 14, 11–21. 10.1016/J.DEVCEL.2007.12.008 18194649 PMC3549671

[B200] Sarkozy A. Carta C. Moretti S. Zampino G. Digilio M. C. Pantaleoni F. (2009). Germline BRAF mutations in noonan, LEOPARD, and cardiofaciocutaneous syndromes: molecular diversity and associated phenotypic spectrum. Hum. Mutat. 30, 695–702. 10.1002/HUMU.20955 19206169 PMC4028130

[B201] Scheibner K. Schirge S. Burtscher I. Büttner M. Sterr M. Yang D. (2021). Epithelial cell plasticity drives endoderm formation during gastrulation. Nat. Cell Biol. 23, 692–703. 10.1038/s41556-021-00694-x 34168324 PMC8277579

[B202] Schonfeldova B. Ai Z. Miotla-Zarebska J. Alraies Z. Magalhaes Pinto M. Malengier-Devlies B. (2025). Aquaporin 1 is a novel mechano-osmotic sensor controlling joint macrophage specification. 10.2139/SSRN.5145345

[B203] Schyrr F. Alonso-Calleja A. Vijaykumar A. Sordet-Dessimoz J. Gebhard S. Sarkis R. (2024). Inducible CXCL12/CXCR4-dependent extramedullary hematopoietic niches in the adrenal gland. Blood 144, 964–976. 10.1182/BLOOD.2023020875 38728427

[B204] Sha M. Lee X. Li X. Veldman G. M. Finnerty H. Racie L. (2000). Syncytin is a captive retroviral envelope protein involved in human placental morphogenesis. Nature 403, 785–789. 10.1038/35001608 10693809

[B205] Shabo I. Svanvik J. Lindström A. Lechertier T. Trabulo S. Hulit J. (2020). Roles of cell fusion, hybridization and polyploid cell formation in cancer metastasis. World J. Clin. Oncol. 11, 121–135. 10.5306/WJCO.V11.I3.121 32257843 PMC7103524

[B206] Sharma D. Bhartiya D. (2021). Stem cells in adult mice ovaries form germ cell nests, undergo meiosis, neo-oogenesis and follicle assembly on regular basis during estrus cycle. Stem Cell Rev. Rep. 17, 1695–1711. 10.1007/S12015-021-10237-4 34455541

[B207] Shimono Y. Zabala M. Cho R. W. Lobo N. Dalerba P. Qian D. (2009). Downregulation of miRNA-200c links breast cancer stem cells with normal stem cells. Cell 138, 592–603. 10.1016/j.cell.2009.07.011 19665978 PMC2731699

[B208] Shin D. M. Liu R. Klich I. Wu W. Ratajczak J. Kucia M. (2010). Molecular signature of adult bone marrow-purified very small embryonic-like stem cells supports their developmental epiblast/germ line origin. Leukemia 24, 1450–1461. 10.1038/LEU.2010.121 20508611

[B209] Shvartsur A. Bonavida B. (2015). Trop2 and its overexpression in cancers: regulation and clinical/therapeutic implications. Genes Cancer 6, 84–105. 10.18632/GENESANDCANCER.40 26000093 PMC4426947

[B210] Simpson A. J. G. Caballero O. L. Jungbluth A. Chen Y. T. Old L. J. (2005). Cancer/testis antigens, gametogenesis and cancer. Nat. Rev. Cancer 5, 615–625. 10.1038/NRC1669 16034368

[B211] Smith A. A. Nip Y. Bennett S. R. Hamm D. C. Lemmers R. J. L. F. van der Vliet P. J. (2023). DUX4 expression in cancer induces a metastable early embryonic totipotent program. Cell Rep. 42, 113114. 10.1016/J.CELREP.2023.113114 37691147 PMC10578318

[B212] Snider L. Geng L. N. Lemmers R. J. L. F. Kyba M. Ware C. B. Nelson A. M. (2010). Facioscapulohumeral dystrophy: incomplete suppression of a retrotransposed gene. PLoS Genet. 6, e1001181. 10.1371/JOURNAL.PGEN.1001181 21060811 PMC2965761

[B213] Sohawon D. Lau K. K. Lau T. Bowden D. K. (2012). Extra-medullary haematopoiesis: a pictorial review of its typical and atypical locations. J. Med. Imaging Radiat. Oncol. 56, 538–544. 10.1111/J.1754-9485.2012.02397.X 23043573

[B214] Sou I. F. Hamer G. Tee W. W. Vader G. McClurg U. L. (2023). Cancer and meiotic gene expression: two sides of the same coin? Curr. Top. Dev. Biol. 151, 43–68. 10.1016/BS.CTDB.2022.06.002 36681477

[B215] Soygur B. Sati L. (2016). The role of syncytins in human reproduction and reproductive organ cancers. Reproduction 152, R167–R178. 10.1530/REP-16-0031 27486264

[B216] Spandidos Publications (2025a). Cell fusion in cancer hallmarks: current research status and future indications. 10.3892/ol.2021.12791 PMC8138896 34055095

[B217] Spandidos-Publications (2025b). “Ovarian endometriosis, a precursor of ovarian cancer: histological aspects,” in Gene expression and microRNA alterations. 10.3892/etm.2021.9674 PMC7851621 33603851

[B218] Stallock J. Molyneaux K. Schaible K. Knudson C. M. Wylie C. (2003). The pro-apoptotic gene Bax is required for the death of ectopic primordial germ cells during their migration in the mouse embryo. Development 130, 6589–6597. 10.1242/DEV.00898 14660547

[B219] Steinert R. Hantschick M. Vieth M. Gastinger I. Kühnel F. Lippert H. (2008). Influence of subclinical tumor spreading on survival after curative surgery for colorectal cancer. Arch. Surg. 143, 122–128. 10.1001/ARCHSURG.2007.49 18283136

[B220] Stephens P. J. Greenman C. D. Fu B. Yang F. Bignell G. R. Mudie L. J. (2011). Massive genomic rearrangement acquired in a single catastrophic event during cancer development. Cell 144, 27–40. 10.1016/j.cell.2010.11.055 21215367 PMC3065307

[B221] Sugatani T. Hruska K. A. (2007). MicroRNA-223 is a key factor in osteoclast differentiation. J. Cell. Biochem. 101, 996–999. 10.1002/JCB.21335 17471500

[B222] Takebe N. Miele L. Harris P. J. Jeong W. Bando H. Kahn M. (2015). Targeting notch, hedgehog, and Wnt pathways in cancer stem cells: clinical update. Nat. Rev. Clin. Oncol. 12, 445–464. 10.1038/NRCLINONC.2015.61 25850553 PMC4520755

[B223] Tang F. Kaneda M. O’Carroll D. Hajkova P. Barton S. C. Sun Y. A. (2007). Maternal microRNAs are essential for mouse zygotic development. Genes Dev. 21, 644–648. 10.1101/GAD.418707 17369397 PMC1820938

[B224] Tang Y. Lu Y. Chen Y. Luo L. Cai L. Peng B. (2019). Pre-metastatic niche triggers SDF-1/CXCR4 axis and promotes organ colonisation by hepatocellular circulating tumour cells *via* downregulation of Prrx1. J. Exp. Clin. Cancer Res. 38, 1–13. 10.1186/S13046-019-1475-6/FIGURES/6 31752959 PMC6873584

[B225] Theunissen T. W. Friedli M. He Y. Planet E. O’Neil R. C. Markoulaki S. (2016). Molecular criteria for defining the naive human pluripotent state. Cell Stem Cell 19, 502–515. 10.1016/J.STEM.2016.06.011 27424783 PMC5065525

[B226] Thul P. J. Akesson L. Wiking M. Mahdessian D. Geladaki A. Ait Blal H. (2017). A subcellular map of the human proteome. Science 356, 356. 10.1126/SCIENCE.AAL3321;PAGEGROUP:STRING:PUBLICATION 28495876

[B227] Tian X. C. Kubota C. Enright B. Yang X. (2003). Cloning animals by somatic cell nuclear transfer – biological factors. Reprod. Biol. Endocrinol. 1, 98. 10.1186/1477-7827-1-98 14614770 PMC521203

[B228] Trosko J. E. (2021). On the potential origin and characteristics of cancer stem cells. Carcinogenesis 42, 905–912. 10.1093/CARCIN/BGAB042 34014276

[B229] Uhlén M. Fagerberg L. Hallström B. M. Lindskog C. Oksvold P. Mardinoglu A. (2015). Tissue-based map of the human proteome. Science, 347 (6220). 10.1126/SCIENCE.1260419 25613900

[B230] Uribe P. Wistuba I. I. González S. (2003). BRAF mutation: a frequent event in benign, atypical, and malignant melanocytic lesions of the skin. Am. J. Dermatopathol. 25, 365–370. 10.1097/00000372-200310000-00001 14501284

[B231] van Furth R. (1998). Mononuclear phagocyte system. Encycl. Immunol., 1755–1758. 10.1006/RWEI.1999.0444

[B232] Vassilopoulos G. Wang P. R. Russell D. W. (2003). Transplanted bone marrow regenerates liver by cell fusion. Nature 422, 901–904. 10.1038/nature01539 12665833

[B233] Ventura A. Young A. G. Winslow M. M. Lintault L. Meissner A. Erkeland S. J. (2008). Targeted deletion reveals essential and overlapping functions of the miR-17∼92 family of miRNA clusters. Cell 132, 875–886. 10.1016/J.CELL.2008.02.019 18329372 PMC2323338

[B234] Vinogradov A. E. Anatskaya O. V. (2025). “Cell dedifferentiation” *versus* “evolutionary reversal” theories of cancer: the direct contest of transcriptomic features. Int. J. Cancer 156, 1802–1813. 10.1002/IJC.35352 39888036

[B235] Vogelstein B. Kinzler K. W. (2004). Cancer genes and the pathways they control. Nat. Med. 10, 789–799. 10.1038/NM1087 15286780

[B236] Walsh C. P. Chaillet J. R. Bestor T. H. (1998). Transcription of IAP endogenous retroviruses is constrained by cytosine methylation. Nat. Genet. 20, 116–117. 10.1038/2413 9771701

[B237] Wang H. Dey S. K. (2006). Roadmap to embryo implantation: clues from mouse models. Nat. Rev. Genet. 73 (7), 185–199. 10.1038/nrg1808 16485018

[B238] Wang X. Cho S. Y. Hu C. S. Chen D. Roboz J. Hoffman R. (2014). CXCL12 influences the development of extramedullary hematopoiesis in the spleens of myelofibrosis patients. Exp. Hematol. 43, 100–109. 10.1016/J.EXPHEM.2014.10.013 25461253 PMC4324010

[B239] Wang C. Gu Y. Zhang K. Xie K. Zhu M. Dai N. (2016a). Systematic identification of genes with a cancer-testis expression pattern in 19 cancer types. Nat. Commun. 7, 10499. 10.1038/NCOMMS10499 26813108 PMC4737856

[B240] Wang R. Chen S. Li C. Ng K. T. P. Kong C. Cheng J. (2016b). Fusion with stem cell makes the hepatocellular carcinoma cells similar to liver tumor-initiating cells. BMC Cancer 16, 1–9. 10.1186/S12885-016-2094-7/FIGURES/7 26846780 PMC4743091

[B241] Wang Y. Wang L. Chen C. Chu X. (2018). New insights into the regulatory role of microRNA in tumor angiogenesis and clinical implications. Mol. Cancer 17, 22. 10.1186/S12943-018-0766-4 29415727 PMC5804051

[B242] Wang B. Yang F. Jin C. Hu J. Qi J. Zhang Q. (2022). Migration of primordial germ cells is regulated by miR-430 during embryonic development of Japanese flounder. Front. Mar. Sci. 9, 904376. 10.3389/FMARS.2022.904376/BIBTEX

[B243] Wang Q. Shi Y. Bian Q. Zhang N. Wang M. Wang J. (2023). Molecular mechanisms of syncytin-1 in tumors and placental development related diseases. Discov. Oncol. 14, 104. 10.1007/S12672-023-00702-6 37326913 PMC10275825

[B244] Wang B. Han J. Elisseeff J. H. Demaria M. (2024). The senescence-associated secretory phenotype and its physiological and pathological implications. Nat. Rev. Mol. Cell Biol. 25, 958–978. 10.1038/s41580-024-00727-x 38654098

[B245] Williams B. R. Amon A. (2009). Aneuploidy: cancer’s fatal flaw? Cancer Res. 69, 5289–5291. 10.1158/0008-5472 19549887 PMC2917070

[B246] Wolf K. Wu Y. I. Liu Y. Geiger J. Tam E. Overall C. (2007). Multi-step pericellular proteolysis controls the transition from individual to collective cancer cell invasion. Nat. Cell Biol. 98 (9), 893–904. 10.1038/ncb1616 17618273

[B247] Wu S. Aksoy M. Shi J. Houbaviy H. B. (2014). Evolution of the miR-290–295/miR-371–373 cluster family seed repertoire. PLoS One 9, e108519. 10.1371/JOURNAL.PONE.0108519 25268927 PMC4182485

[B248] Wu K. Guo C. Li Y. Yang J. Zhou Q. Cheng S. (2021). MicroRNA-18a-5p regulates the warburg effect by targeting hypoxia-inducible factor 1α in the K562/ADM cell line. Exp. Ther. Med. 22, 1069. 10.3892/ETM.2021.10503 34447462 PMC8355681

[B249] Xiang X. Tao Y. DiRusso J. Hsu F. M. Zhang J. Xue Z. (2022). Human reproduction is regulated by retrotransposons derived from ancient Hominidae-specific viral infections. Nat. Commun. 13, 1–15. 10.1038/s41467-022-28105-1 35075135 PMC8786967

[B250] Yamaguchi S. Hong K. Liu R. Shen L. Inoue A. Diep D. (2012). Tet1 controls meiosis by regulating meiotic gene expression. Nature 492, 443–447. 10.1038/NATURE11709 23151479 PMC3528851

[B251] Yamanaka S. Blau H. M. (2010). Nuclear reprogramming to a pluripotent state by three approaches. Nature 465, 704–712. 10.1038/nature09229 20535199 PMC2901154

[B252] Yang Y. Zheng H. Zhan Y. Fan S. (2019). An emerging tumor invasion mechanism about the collective cell migration. Am. J. Transl. Res. 11, 5301–5312. 31632511 PMC6789225

[B253] Yao G. D. Shu Y. M. Shi S. L. Peng Z. F. Song W. Y. Jin H. X. (2014). Expression and potential roles of HLA-G in human spermatogenesis and early embryonic development. PLoS One 9, e92889. 10.1371/JOURNAL.PONE.0092889 24667226 PMC3965489

[B254] Yasui M. Yamamoto H. Ngan C. Y. Damdinsuren B. Sugita Y. Fukunaga H. (2006). Antisense to cyclin D1 inhibits vascular endothelial growth factor–stimulated growth of vascular endothelial cells: implication of tumor vascularization. Clin. Cancer Res. 12, 4720–4729. 10.1158/1078-0432.CCR-05-1213 16899623

[B255] Yuan K. Ai W. B. Wan L. Y. Tan X. Wu J. F. (2017). The miR-290-295 cluster as multi-faceted players in mouse embryonic stem cells. Cell Biosci. 7, 1–10. 10.1186/S13578-017-0166-2/FIGURES/2 28794853 PMC5547456

[B256] Zack T. I. Schumacher S. E. Carter S. L. Cherniack A. D. Saksena G. Tabak B. (2013). Pan-cancer patterns of somatic copy number alteration. Nat. Genet. 45, 1134–1140. 10.1038/ng.2760 24071852 PMC3966983

[B257] Zhang Y. Xue Y. Cao C. Huang J. Hong Q. Hai T. (2017). Thyroid hormone regulates hematopoiesis *via* the TR-KLF9 axis. Blood 130, 2161–2170. 10.1182/BLOOD-2017-05-783043 28972010

[B258] Zhang S. Zhou K. Luo X. Li L. Tu H. C. Sehgal A. (2018). The polyploid state plays a tumor-suppressive role in the liver. Dev. Cell 44, 447–459.e5. 10.1016/J.DEVCEL.2018.01.010 29429824 PMC5828993

[B259] Zhang F. Liu R. Liu C. Zhang H. Lu Y. (2020a). Nanos3, a cancer-germline gene, promotes cell proliferation, migration, chemoresistance, and invasion of human glioblastoma. Cancer Cell Int. 20, 197. 10.1186/S12935-020-01272-1 32508533 PMC7249350

[B260] Zhang F. Liu R. Zhang H. Liu C. Liu C. Lu Y. (2020b). Suppressing dazl modulates tumorigenicity and stemness in human glioblastoma cells. BMC Cancer 20, 673. 10.1186/S12885-020-07155-Y 32682409 PMC7368788

[B261] Zhao B. C. Wang Z. J. Mao W. Z. Ma H. C. Han J. G. Zhao B. (2011). CXCR4/SDF-1 axis is involved in lymph node metastasis of gastric carcinoma. World J. Gastroenterol. 17, 2389–2396. 10.3748/WJG.V17.I19.2389 21633638 PMC3103791

[B262] Zhao B. Wu Q. Ye A. Y. Guo J. Zheng X. Yang X. (2019). Somatic LINE-1 retrotransposition in cortical neurons and non-brain tissues of rett patients and healthy individuals. PLoS Genet. 15, e1008043. 10.1371/JOURNAL.PGEN.1008043 30973874 PMC6478352

[B263] Zhou L. Dean J. (2014). Reprogramming the genome to totipotency in mouse embryos. Trends Cell Biol. 25, 82–91. 10.1016/J.TCB.2014.09.006 25448353 PMC4312727

[B264] Zhou S. Sakashita A. Yuan S. Namekawa S. H. (2022). Retrotransposons in the mammalian Male germline. Sex. Dev. 16, 404–422. 10.1159/000520683 35231923 PMC11974347

[B265] Zhou L. Le M. N. U. Du Y. Chen X. Jin M. Xiang H. (2024). A novel cancer-germline gene DAZL promotes progression and cisplatin resistance of non-small cell lung cancer by upregulating JAK2 and MCM8. Gene 916, 148449. 10.1016/J.GENE.2024.148449 38588931

[B266] Zhu X. Fang H. Gladysz K. Barbour J. A. Wong J. W. H. (2021). Overexpression of transposable elements is associated with immune evasion and poor outcome in colorectal cancer. Eur. J. Cancer 157, 94–107. 10.1016/j.ejca.2021.08.003 34492588

[B267] Zwaka T. P. Thomson J. A. (2005). A germ cell origin of embryonic stem cells? Development 132, 227–233. 10.1242/DEV.01586 15623802

